# Comparison of serum and saliva miRNAs for identification and characterization of mTBI in adult mixed martial arts fighters

**DOI:** 10.1371/journal.pone.0207785

**Published:** 2019-01-02

**Authors:** Daria LaRocca, Sarah Barns, Steven D. Hicks, Andrew Brindle, Jeremy Williams, Richard Uhlig, Paul Johnson, Christopher Neville, Frank A. Middleton

**Affiliations:** 1 Department of Neuroscience & Physiology, SUNY Upstate Medical University, Syracuse, NY United States of America; 2 Quadrant Biosciences, Inc., 405 Irving Avenue, Syracuse, NY, United States of America; 3 Department of Pediatrics, Penn State College of Medicine, Hershey, PA, United States of America; 4 College of Health Professions—Clinical Laboratory Science, SUNY Upstate Medical University, Syracuse, NY, United States of America; 5 Department of Physical Therapy Education, SUNY Upstate Medical University, Syracuse, NY, United States of America; 6 Department of Psychiatry & Behavioral Sciences, SUNY Upstate Medical University, Syracuse, NY, United States of America; 7 Department of Biochemistry & Molecular Biology, SUNY Upstate Medical University, Syracuse, NY, United States of America; 8 Department of Pediatrics, SUNY Upstate Medical University, Syracuse, NY, United States of America; Kunming University of Science and Technology, CHINA

## Abstract

Traumatic brain injury (TBI) is a major cause of death and disability worldwide, with mild TBI (mTBI) accounting for 85% of cases. mTBI is also implicated in serious long-term sequelae including second impact syndrome and chronic traumatic encephalopathy. mTBI often goes undiagnosed due to delayed symptom onset and limited sensitivity of conventional assessment measures compared with severe TBI. Current efforts seek to identify accurate and reliable non-invasive biomarkers associated with functional measures relevant to long-term outcomes. Here we evaluated the utility of serum and salivary microRNAs (miRNAs) to serve as sensitive and specific peripheral biomarkers of possible mTBI. Our primary objectives were to establish the relationship between peripheral measures of miRNA, objective quantification of head impacts, and sensitive indices of balance and cognitive function in healthy young adult athletes. A secondary objective was to compare the sensitivity of miRNA versus commonly used blood-based protein biomarkers. 50 amateur mixed martial arts (MMA) fighters participated. 216 saliva and serum samples were collected at multiple time points, both pre- and post-fight. Levels of 10 serum proteins were compared in a subset of the fighters (n = 24). Levels of miRNAs were obtained by next generation sequencing. Functional outcomes were evaluated using a computerized assessment system that measured cognitive performance, body sway, and combined cognitive performance and body sway during dual task completion. Data were analyzed using multivariate logistic regression for predictive classification, analysis of variance, correlation analysis and principal component analysis. We identified a subset of salivary and serum miRNAs that showed robust utility at predicting TBI likelihood and demonstrated quantitative associations with head impacts as well as cognitive and balance measures. In contrast, serum proteins demonstrated far less utility. We also found that the timing of the responses varies in saliva and serum, which is a critical observation for biomarker studies to consider.

## Introduction

Traumatic brain injury (TBI) is an important public health problem, affecting at least 1.7 million individuals annually in the U.S. alone [1], and is predicted to "surpass many diseases as the major cause of death and disability by the year 2020" according to the WHO [2]. The disorder is classified on a spectrum ranging from mild to severe, with mild TBI (mTBI) accounting for at least 85% of total TBI cases [3]. Notably, the incidence of mTBI is commonly regarded as under-reported, particularly in the context of sports competitions, where athletes may wish to avoid being forced to stop participation until completion of a formal medical evaluation and return to play protocol. As a result, mTBI has been referred to as a “silent epidemic” [4].

The typical head impact in mTBI induces rapid percussive (coup/contracoup) and/or torsional (rotational) damage to the brain, leading to parenchymal bruising and subarachnoid hemorrhage with direct brain cell loss, as well as stretching of axons and diffuse axonal injury [5] that may persist for years [6]. Furthermore, repetitive mTBI is associated with serious long-term sequelae including post concussive syndrome and chronic traumatic encephalopathy (CTE), the latter often leading to cognitive impairment, neuropsychiatric symptoms, dementia, and pugilistic parkinsonism. Because these symptoms develop across time and the initial injuries often escape detection by conventional neuroimaging techniques, mTBI presents a diagnostic challenge, which has slowed efforts to examine the time course of its pathophysiology. Consequently, diagnostic, prognostic, and therapeutic approaches for mTBI are lacking. Compounding this issue, the failure to ascertain that mTBI has occurred in the first place can easily lead to repetitive mTBI and increase the risk of CTE. Thus, it is critically important to establish accurate and reliable diagnostic markers to aid in the early detection and diagnosis of mTBI, inform its prognosis, and ultimately provide a means to monitor response to treatment.

MicroRNAs (miRNA) are small non-coding RNAs (~22 nucleotides) that suppress target mRNA translation and stability [7] for a large fraction of the transcriptome, and have emerged as useful biomarkers of several disorders including cancer and diabetes. The influence of miRNAs on gene expression occurs both within the cells that synthesize them as well as within remote cells through extracellular trafficking. Once released from donor cells, miRNAs can travel through various extracellular fluids and exert regulatory effects on gene expression in recipient cells. Hence, miRNAs are important master regulators of cellular function within and between a wide range of cells and tissues. Recent data indicating that circulating miRNAs are elevated in plasma following injury [8], and that miRNA expression profiles differ between healthy and disease states, has generated considerable interest in their potential to serve as peripheral biomarkers of cell and tissue damage or cancer (reviewed in [9]). In addition, dysregulation of specific miRNAs networks has been associated with several neurodegenerative disorders including Alzheimer's and Parkinson's disease (reviewed in [10]), as well as alcoholism [11]. While brain tissue is not readily available from living subjects with mTBI or neurodegenerative disease, the fact that brain-specific miRNAs are released into peripheral biofluids suggests that miRNA profiles can serve as a proxy, or indirect readout of pathological processes occurring in the central nervous system (CNS). Thus, identifying specific biomarkers for mTBI could facilitate early detection at the presymptomatic stage, and provide insight into novel targets to minimize or even prevent post-mTBI sequelae. Support for the feasibility of using peripheral miRNA biomarkers to predict outcome measures following mTBI was recently provided in two studies on pediatric populations. The first study demonstrated considerable overlap in the miRNA present in both cerebrospinal fluid (CSF) and saliva (63%), and also indicated parallel changes for a number of these miRNAs in children with severe and mild TBI [12]. A follow up study from the same group showed that salivary miRNA patterns in children who were brought to a concussion clinic within a few days after mTBI could predict whether those children would develop prolonged concussive symptoms (PCS) or acute symptom resolution with high accuracy [13].

Notably, one of the elements missing from the aforementioned studies is any type of molecular or functional baseline assessment in the individuals that subsequently experienced an mTBI episode. This was specifically addressed in the present study, where we directly compare the pattern of changes in saliva and serum miRNAs, and changes in numerous neurocognitive functional measures in adult athletes after they experienced sport-related head impacts and possible mTBI during an amateur mixed martial arts (MMA) competition. Furthermore, we quantified the strength of association between the changes in miRNAs and functional measures, and assessed their potential diagnostic utility.

## Materials and methods

### Ethics statement

All protocols regarding the use of human subjects were reviewed and approved by the Institutional Review Board of SUNY Upstate Medical University. Written consent was obtained from all human subjects prior to study enrollment and sample collection. Subjects received monetary compensation for their participation.

### Samples

**Fig 1:**
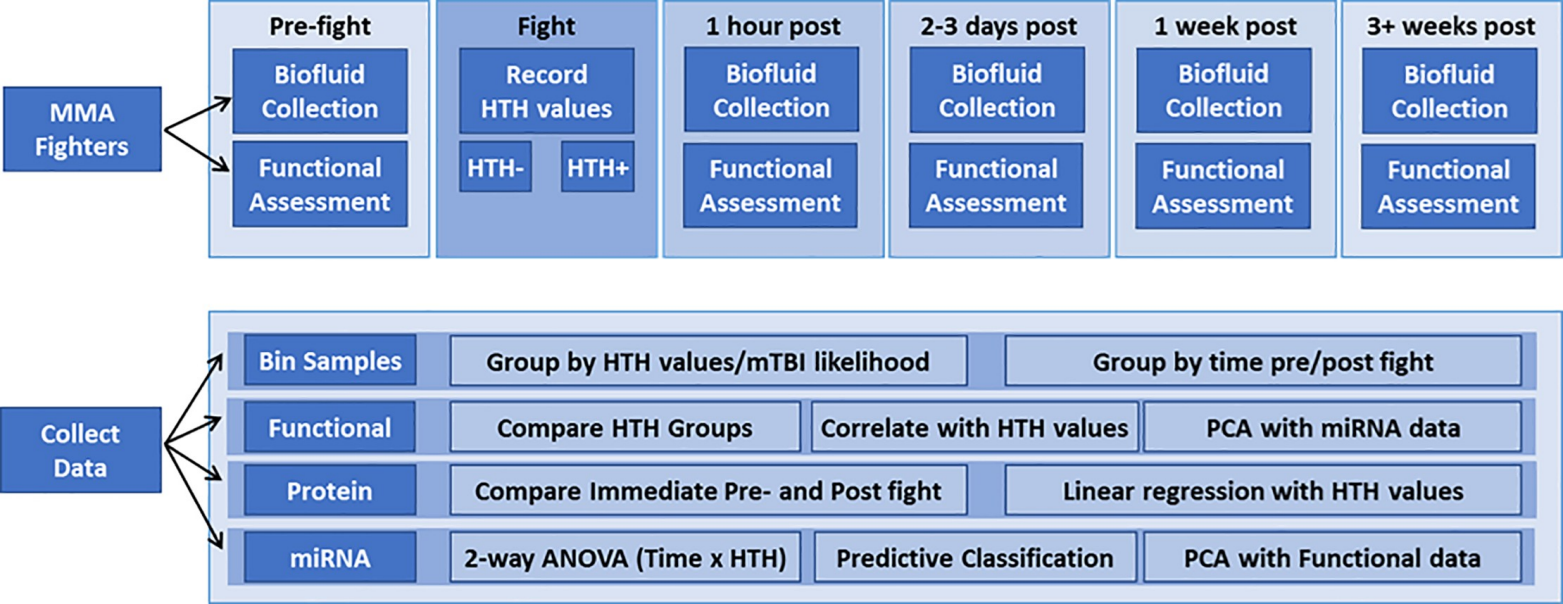
Samples and analyses in the present study. HTH, Hits to the Head; PCA, principal component analysis.

A total of 218 samples were collected from 50 amateur MMA fights (42 unique, 8 repeat fighters), including 87 saliva and 131 serum samples. These were collected at 1 week or 1 hour pre-fight time points, and at one or more of 4 post-fight time points: immediately post-fight (15–30 min), 2–3 days, 1 week, and 3+ weeks (**Fig 1**, **Table 1**). Each MMA fight consisted of three rounds of 3 minutes each, unless a fighter was knocked out or forfeited by submission. Blood collection was performed on-site by a trained phlebotomist into sterile BD Vacutainer SST tubes (Becton-Dickenson), allowed to sit for 20 minutes and centrifuged per manufacturer instructions. Saliva was collected by expectoration into Oragene RNA collection vials (RE-100, DNA Genotek, Ottawa, ON) or by swab absorption using the Oragene Nucleic Acid Stabilizing Kit swab (P-157, DNA Genotek, Ottawa, ON).

**Table 1:** Saliva and serum samples used for miRNA analysis.

	N	1 wk pre	0 d pre	0 d post	2–3 d post	1 wk post	3+ wks post	Functional Data	
**Saliva.**	87	4	23	26	15	12	7	54	64%
**Serum.**	131	7	52	52	17	3	0	49	37%
**Total.**	218	11	75	78	32	15	7	103	48%

The MMA subjects included 40 males and 2 females, with an average age of 26.5 yrs (SD ±5.8) and mean BMI of 24.6 (SD ±3.3). Twenty-eight (66%) of the subjects self-reported as Caucasian, 7 (17%) African American, and 5 (14%) Hispanic. A total of 12 (29%) of the fighters also reported a prior history of concussion, without complication. Serum samples from a subset of these fighters were used to evaluate potential changes in pre- and post-fight protein biomarkers of mTBI. These samples were derived from 24 fighters (23 male), aged 18–42 (mean 24.9 yrs), with a mean BMI of 23.4. One of the subjects had a noted history of hearing loss, and 5 had a previous history of a single concussion (without complication). The majority (57%) of these fighters were Caucasian, 20% were African American, and 20% were Hispanic.

### Functional studies

Assessment of MMA fighter balance and cognitive function was performed using a version of the ClearEdge assessment system developed by Quadrant Biosciences Inc. (Syracuse NY). ClearEdge is a Class I medical device approved by the FDA that measures body sway in three dimensions during 8 different stances, as well as body sway and completion times during the performance of dual motor and cognitive tasks. The dual tasks and cognitive tasks were completed by each subject using a hand-held tablet computer (Toshiba, Model: WTB-B) and stylus. The analysis of body sway (balance) was measured via the use of an inertial sensor worn by each subject around the waist that sampled motion in all three planes at a frequency of 250 Hz with the resulting data downloaded from each tablet for post-processing. Stances were held by each subject for 30 seconds, with their shoes removed, while standing either on the floor or on a foam pad and data were obtained with the eyes open or closed. During the stances, the feet were either positioned side by side with the ankles or medial aspects of the feet touching, or they were in a tandem position with the dominant foot forward and the non-dominant foot positioned directly behind and the heel of the lead foot in contact with the toes of the trailing foot. The cognitive component of the dual tasks included a digital version of the Trails A and Trails B tasks, and an auditory working memory task (Backward Digit Span) in addition to a simple dual task of merely holding the tablet steady while maintaining fixation on it. In Trails A, subjects had to quickly connect an ascending series of encircled numbers (1-2-3 etc.) with a stylus on the screen. In Trails B, subjects had to connect an ascending series of encircled numbers and letters in an alternating alpha-numeric sequence (1-A-2-B-3-C etc.). The Backward Digit Span task consisted of measuring reverse-order auditory recall of increasingly long number sequences that were delivered to each subject via headphones. Altogether, 14 tasks were measured on the fighters (**Table 2**). Notably, it was only possible to obtain simultaneous functional and biofluid measures on the same subjects in approximately half (48%) of the sample times (**Table 1**).

**Table 2:** Functional outcome measures.

**Standing on floor**	
1)	Sway during Two Legs Eyes Open (TLEO)
2)	Sway during Two Legs Eyes Closed (TLEC)
3)	Sway during Tandem Stance Eyes Open (TSEO)
4)	Sway during Tandem Stance Eyes Closed (TSEC)
**Standing on foam pad**	
5)	Sway during TLEO Foam Pad (TLEOFP)
6)	Sway during TLEC Foam Pad (TLECFP)
7)	Sway during TSEO Foam Pad (TSEOFP)
8)	Sway during TSEC Foam Pad (TSECFP)
**Dual task**	
9)	Sway during Holding Tablet (HT)
10)	Sway during Dual Task Trails B Task (TMB_Dual_Bal)
11)	Sway during Dual Task Digit Span Backwards (DSB_Bal)
12)	Completion Time for Trails A Task (TMA_Cog)
13)	Completion Time for Trails B Task (TMB_Cog)
14)	Completion Time for Dual Task Digit Span Backwards (DSB_Cog)

#### Subject binning

A prior study of more than 840 professional MMA matches established that the likelihood of a fighter experiencing a technical knockout (which would be consistent with concussion symptoms) was related to the number of hits to the head (HTH) they received [14]. Thus, we binned our subjects based on the likelihood of a sports-related concussion consistent with mTBI based on HTH levels. Samples were split into 3 groups based on HTH scores obtained from video recordings and defined by their probability of mTBI as Low (0–3 HTH; mean = 0.3), Moderate (4–9 HTH; mean = 6.5), and Very Likely (10+ HTH; mean = 24.2) (**Table 3)**.

**Table 3:** Sample classifications used in analysis, separated by fluid type.

Comparison Types by mTBI Risk (HTH)		Sample N	Fluid Type	Ave HTH
Low	0–3 HTH	50	24 saliva 26 serum	0.3
Moderate	4–9 HTH	41	15 saliva 26 serum	6.5
Very Likely	10–65 HTH	50	23 saliva 27 serum	24.2

HTH, Hits to the head (observed by video)

#### Statistical analysis of functional data

Functional data were converted to standardized difference measures by comparison of all post-fight timepoints with a common pre-fight timepoint within each subject. Missing data points for some of the Backward Digit Span task measures were filled in using a K-nearest neighbor approach. The normalized functional data were screened for sphericity prior to statistical analysis using principal component analysis (PCA). Then, a two-way analysis of variance (ANOVA) was performed to screen for functional measures with a significant effect of the TBI risk classification assignment at the time of collection, while controlling for the biofluid type, with the False Discovery Rate (FDR) < 0.05. We also examined the relationships of the significantly changed functional parameters with each other using Pearson’s correlation metric and an R to T test of significance.

### Protein biomarkers in serum

On a subset (n = 24) of the fighters for whom we had blood samples available immediately pre- and post-fight, we examined expression of several candidate protein biomarkers of TBI based on pre-existing literature (which often focused on severe TBI cases or animal models) using an ELISA or Luminex platform, with aliquots taken from the same vial and stored at -80°C for subsequent processing.

#### Luminex assay

Using a custom 8-plex Magnetic Luminex Screening Panel (R&D Systems, Minneapolis, MN; catalog # LXSAHM), serum samples were assayed for the expression level of BDNF, CCL2/MCP-1, CRP, ICAM1, IL-6, NSE2, S100B, and VCAM according to the manufacturer's protocol. The sensitivity limits for each analyte were 0.32, 9.9, 116, 140, 87.9, 1.7, 4.34, and 238 pg/mL, respectively. Sample fluorescence was read on a Bio-Rad Bioplex 200 System and analyzed using Bioplex Manager 6.1 software (Bio-Rad, Hercules, CA).

#### Enzyme Linked Immunosorbent Assay (ELISA)

Serum levels of UCHL1, MBP, GFAP were detected using Mybiosource ELISA kits (MyBiosource, Inc., San Diego, CA) according to the manufacturer's instructions. The catalog numbers and detection limits were as follows: UCHL1 (# MBS2512760), 78.125-5000pg/mL; MBP (#MBS261463), 15.6 pg/ml-1000 pg/ml; and GFAP (#MBS262801), 0.312 ng/ml-20 ng/ml. The optical density of the peroxidase product was measured spectrophotometrically using a Synergy 2 microplate reader (Biotek, Winooski, VT) at a wavelength of 450 nm.

Statistical analysis of the protein biomarker data was performed using a pairwise T test comparing the post-fight levels to the pre-fight levels for the 24 fighters, as well as linear regression to examine the relationship of the changes in post-fight levels compared to the number of hits to the head (HTH) that were observed from fight videos for each subject.

### Next generation sequencing of serum and saliva RNA

#### RNA isolation

RNA was isolated from serum and saliva using the miRNeasy Serum/Plasma Kit (Qiagen, Inc.) according to the manufacturer's instructions. *Serum*: frozen serum samples were thawed on ice, and 200μL of serum was added to 1mL of QIAzol lysis reagent. Following vigorous vortexing, 200μL of chloroform was added and the samples were incubated for 5 minutes at room temperature (RT), then centrifuged at 12,000 x g for 15 minutes at RT. The resultant aqueous phase was removed, mixed with 1.5 volumes of 100% ethanol, transferred to an RNeasy MinElute spin column, and centrifuged for 15 seconds. The column was washed with Buffers RWT and RPE at the manufacturer's indicated volumes, and the RNA was eluted with 30μL of RNase-free water. *Saliva*: refrigerated saliva samples originally collected in an Oragene vial or swab collection kit were incubated at 50°C for 1 hour. A 250μL aliquot was then removed, transferred to a microcentrifuge tube, incubated at 90°C for 15 minutes, and cooled to RT. 750μL of QIAzol lysis reagent was added, and the sample was vortexed vigorously for 1 minute, and incubated for 5 minutes at RT. Chloroform (200μL) was added, and the sample was vortexed for 1 minute, then centrifuged at maximum speed (>13,000 x g) for 10 minutes. 450μL of the resultant aqueous phase was transferred to a new tube, mixed with 675μL of 100% ethanol, transferred to an RNeasy MinElute spin column, and centrifuged for 15 seconds. The column was sequentially washed with Buffers RWT and RPE at the manufacturer's indicated volumes, and the RNA was eluted with 30μL of RNase-free water. RNA quality was assessed using the Agilent Technologies Bioanalyzer on the RNA Nanochip.

Stranded RNA-sequencing libraries were prepared using the TruSeq Stranded Small RNA Kit (Illumina) according to manufacturer instructions. Samples were indexed in batches of 48, with a targeted sequencing depth of 10 million reads per sample. Sequencing was performed using 36 bp single end reads on an Illumina NextSeq 500 instrument at the SUNY Molecular Analysis Core (SUNYMAC) at Upstate Medical University. FastQ files were trimmed to remove adapter sequences, and alignment performed to the mature miRbase21 database using the Shrimp2 algorithm in Partek Flow (Partek, Inc., St. Louis, MO).

#### RNA-Seq analysis

The aligned reads were quantified and normalized to an internal invariant reference miRNA (miR-24-3p) and converted to log2 scale. As with the functional data, each subject’s normalized miRNA post-fight data was contrasted with their respective pre-fight/baseline values (obtained at either 1 week pre-fight or immediately prior to the fight), yielding a total of 141 sample difference values (n = 62 saliva, 79 serum). These normalized miRNA difference values were screened for sphericity using principal component analysis (PCA) prior to statistical analysis and filtered to eliminate those with more than 60% missingness.

#### Effects of HTH on miRNA levels

We initially used a two-way analysis of variance (ANOVA) to examine the main effects of Sample Type and mTBI Risk (as defined by HTH) as well as their interaction to screen for miRNAs with a significant effect of the mTBI Risk on their expression level. This was performed in all of the samples from both biofluids with a False Discovery Rate (FDR) correction < 0.15. The miRNAs which passed this filter were then used in a stepwise linear regression to establish the miRNAs that best predicted the actual HTH values. A logistic regression classification analysis was then completed to assess the ability to distinguish all of the Very Likely and Low probability TBI samples from each other (holding out the Moderate group). 100-fold Monte-Carlo Cross-Validation (MCCV) was performed to estimate empirical accuracy across biofluids. miRNAs that showed the strongest predictive utility were then subjected to functional analysis using Diana Tools miRpathv3. The difference levels of miRNAs showing strong discriminatory and predictive power were also assessed in relation to various functional measures using Pearson correlation analysis.

#### Effects of time on miRNA levels

Because the first miRNA analysis combined all the initial samples from each subject post-fight into the same TBI probability class, it was possible some miRNAs may have eluded detection if they only had acute or delayed effects at particular time points. Such temporal-dependent responses, if present, could be as meaningful as any derived from the subject binning. To reveal potential acute or delayed effects we used a General Linear Model to examine the effects of Time and Sample Type, and their interaction, on relative miRNA expression based on four different temporal bins. A total of 122 of the samples were used in this analysis. Time 1 contained samples from subjects who showed up to the MMA match but did not participate in a fight, and still provided a biofluid sample (these serve as controls for non-specific effects of the event) as well as subjects that participated in a match but experienced no hits to the head (these serve as exercise controls). Collectively, these are referred to as Time 1 HTH negative (HTH -) Controls. The remaining temporal bins were from fighters who participated in a match and received at least 2 hits to the head (HTH+). These HTH+ samples were grouped by collection time point into Time 1 HTH+ (within 1 hour post-fight), Time 2 HTH+ (2–3 days post-fight), and Time 3 HTH+ (7 days post-fight). The temporal profiles of all miRNAs with significant Time effects were visualized using line plots and subjected to supervised classification analysis to identify the most salient patterns. miRNAs with expression profiles of interest were then subjected to functional analysis using Diana Tools miRpathv3 and compared with the miRNAs from the Subject Binning analysis.

#### Analysis of temporal patterns in functional and miRNA data

After identifying miRNAs with expression profiles of interest, we examined the balance and cognitive score data along with the molecular data using principal component analysis (PCA) to detect the molecular and functional features that show the most similarity across time. For this analysis, only miRNAs with defined patterns (Acute Saliva Response miRNAs or Delayed Serum Response miRNAs) were used with the functional data from all of the post-fight samples (n = 39 saliva, n = 31 serum). Iterative principal axis PCA was performed using a quartimax root curve extraction. Factor weights were examined to identify functional variables most similar to the miRNA variables, with line plots created for visualization purposes.

#### Validation of NGS data

To validate the expression patterns in the NGS-based miRNA data, we elected to use a custom FirePlex assay, which has been shown to perform comparable to qRT-PCR in the validation of RNA sequencing data. A subset of the miRNAs were chosen, including 11 changed and 3 unchanged (5 increased and 3 decreased), along with the reference miRNA used for normalization of the NGS data, and a total of 65 samples (from 63 of the NGS samples with sufficient RNA remaining) were sent to the FirePlex Assay Service lab at AbCam to perform the validation in a blind fashion. Due to low yields in serum, only saliva samples were sent. Included in the samples were two pairs of stabilized saliva for comparison of the signals with purified salivary RNA.

## Results

### Functional changes in MMA fighters

Four of the 14 functional measures showed a significant difference due to TBI likelihood classification, while controlling for the type of biofluid that was being sampled at the time of collection and none showed any interaction effect (**Table 4**). These tasks included three measures of body sway (TLEC, DSB_Bal, TMB_Bal) and one measure of cognitive function (TMA_Cog).

**Table 4:** Significant effects on functional data obtained during biofluid sampling.

Functional Task	TBI	Fluid	Interaction
Digit Span Backwards (Sway)	0.00004	0.84799	0.23975
Two Legs Eyes Closed (Sway)	0.00049	0.84799	0.71747
Trail Making B Dual Task (Sway)	0.02047	0.84799	0.83046
Trail Making A (Cognitive)	0.04340	0.84799	0.83046

P values are FDR-corrected from a 2-way ANOVA

**Fig 2:**
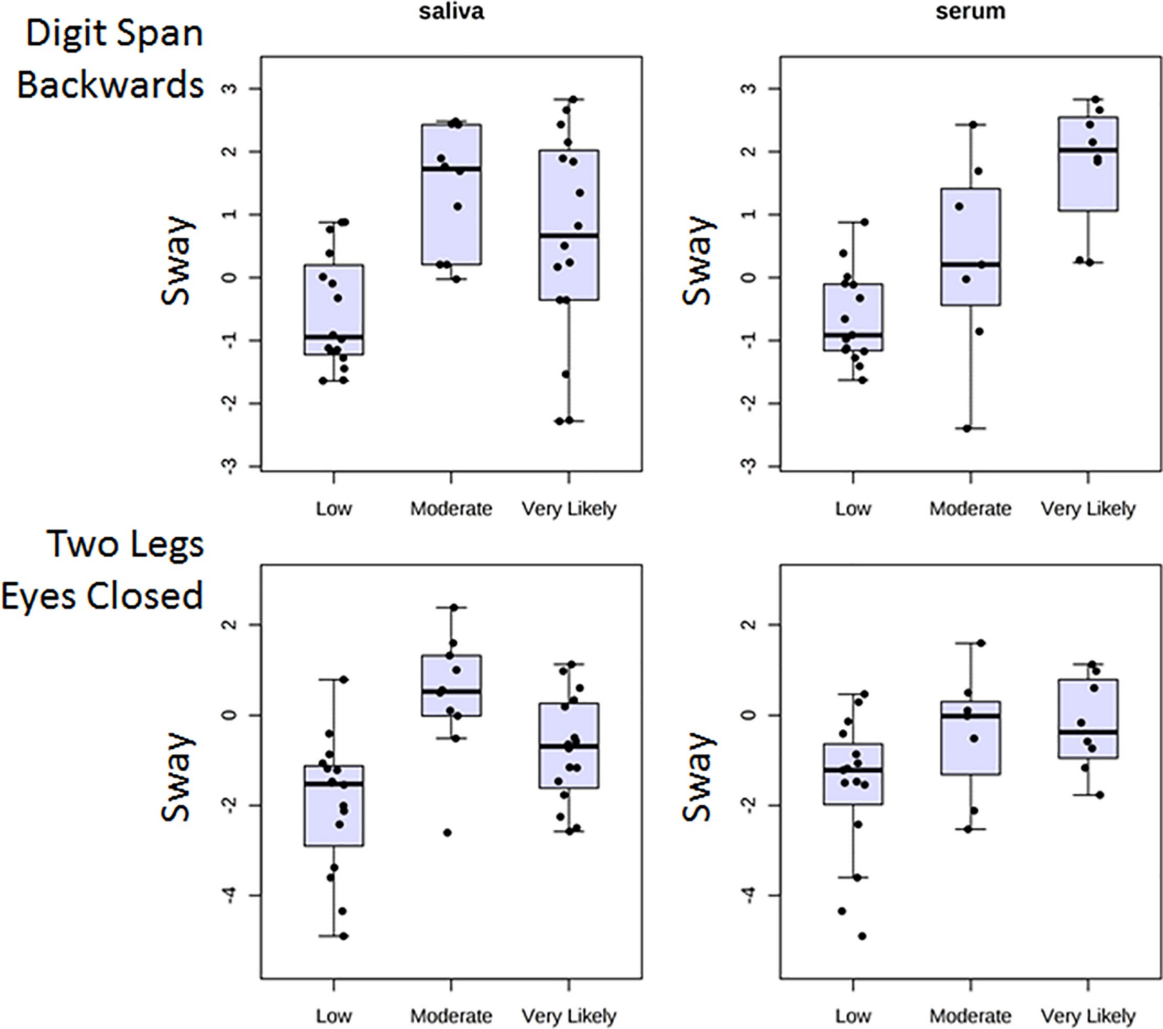
Hits to the head increase postural sway post-fight versus pre-fight. MMA fighters who provided saliva or serum samples were classified into three different TBI likelihood categories (Low, Moderate, Very Likely) based on video recordings. Note that one of the sway measures was obtained during a cognitive task performance (Digit Span Backwards, upper) while the other was obtained during a balance test performed without visual guidance (Two Legs, Eyes Closed, lower). The increase in sway is evident for both sets of measures in the Moderate and Very Likely groups compared with Low TBI likelihood groups.

Although there was no effect of biofluid type, we examined the patterns of functional changes for the sets of subjects providing saliva and serum separately, to help gauge reproducibility. Examples of the patterns of change in the body sway measures during the DSB and TLEC tasks are provided (**Fig 2**). Overall, both of these functional measures increased in the Moderate and Very Likely TBI groups relative to the Low likelihood group. Notably, the patterns were not identical in both subject sample sets because different groups of subjects were assessed, with only partial overlap for 14 of the subjects who provided both saliva and serum along with functional data at the time of collection.

**Fig 3:**
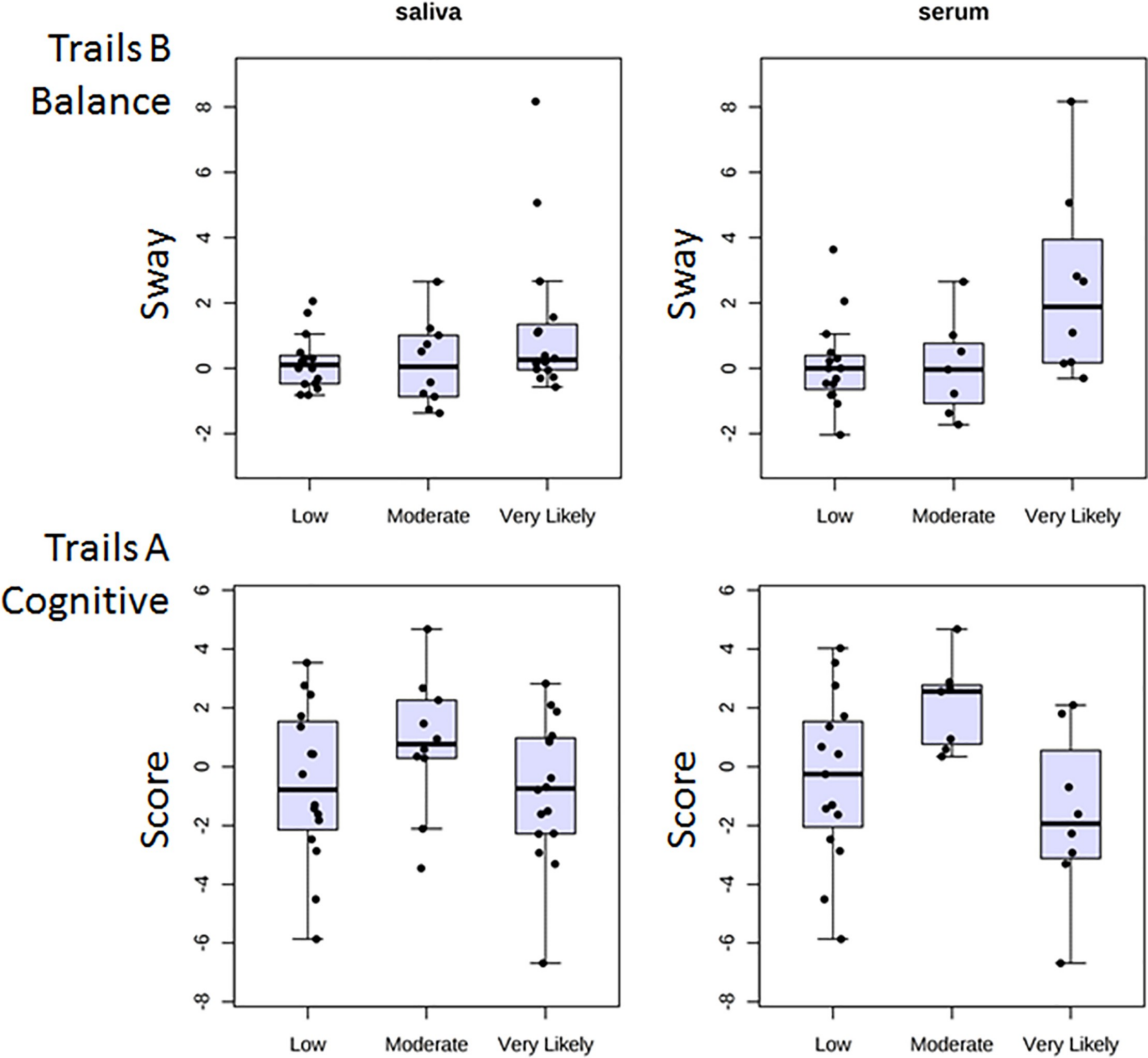
Changes in body sway or completion time scores post-fight are less consistent in two different dual-task functional tests. Subjects are grouped by TBI likelihood. Same conventions as Fig 2. Note slightly elevated scores in the Very Likely group of the TMB_Bal task (upper) when a serum (but not a saliva) sample was taken, and the slight elevation in the TMA_Cog score (lower) in the Moderate (but not Very Likely) group.

In addition to the two functional measures that showed clear stepwise gradients of impairment in the MMA fighters according to probability of TBI, there were two other significantly changed functional measures that did not show as clear a pattern according to TBI likelihood (**Fig 3**). These included the sway during the Trailmaking B task (TMB_Bal) and the difference score of the completion time for the Trailmaking A task (TMA_Cog). For the TMB_Bal task, there was a trend for elevated scores in the Very Likely group, particularly in subjects providing a serum sample, but it was not as evident in the subjects who provided a saliva sample (**Fig 3, top**). For the TMA_Cog task, the pattern was mixed, with a potential elevation in completion time seen in the Moderate group, but no change or a slight decrease in the Very Likely group (**Fig 3, bottom**).

Exploration of the functional changes indicated that difference score measures in body sway during the TLEC task and DSB_Bal tasks were the most strongly associated with TBI likelihood. We thus examined the correlation between these two variables. Using 51 pairs of measures (excluding the missing values replaced by the K-nearest neighbor algorithm) we observed a complete absence of correlation in the two measures (Pearson’s R = 0.00, p = 0.99). Thus, although both tasks are sensitive to differences in balance as a function of the likelihood of TBI (i.e., the hits to the head), they clearly provide different information. However, given the increased difficulty in obtaining Digit Span scores on all subjects because of the need to wear headphones, the TLEC task clearly has practical advantages.

### Serum protein biomarkers

We examined the potential changes in levels of 11 serum proteins in 24 fighters immediately after their fight compared to pre-fight. These proteins included UCHL1, MBP, GFAP (analyzed by ELISA) and BDNF, CCL2/MCP-1, CRP, ICAM1, IL-6, NSE2, S100B, and VCAM (analyzed by a custom Luminex assay. All of the IL-6 sample values were below the lowest standard concentration for that assay, and thus no results were available for this analyte. The majority (21/24) of the S100B values for pre-fight samples were also below the lowest standard concentration. However, 16 of the samples from the same fighters had measurable levels of S100B post-fight. In order to estimate the magnitude of changes and perform statistical comparisons for these 16 samples, we set the pre-fight concentration equal to half the lowest post-fight concentration value (22.7 pg/mL).

**Fig 4:**
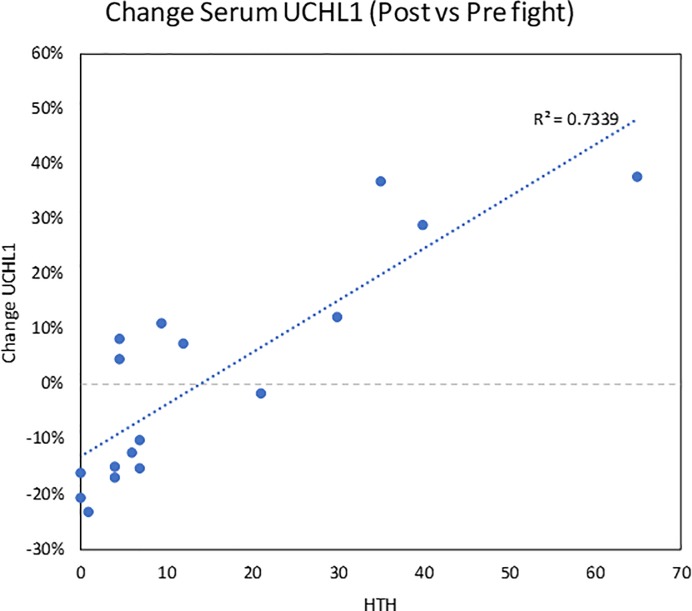
Change in serum UCHL1 post-fight related to hits to the head (HTH). Note that this regression was largely driven by 4 fighters who received more than 30 HTH. Overall, however, there was no significant difference in the group of fighters post-fight versus pre-fight.

Of the 10 proteins we obtained concentrations for, four demonstrated significant pairwise changes (all increases) in post-fight versus pre-fight serum samples. These included GFAP (p = 1.4e-7, median % change = 33.1%), MBP (p = 0.003, median % change = 65.4), NSE2 (p = 0.037, median % change = 50.4), and S100B (p = 0.006, median % change = 747%). Notably, however, the mean increases were present across all HTH levels. To further explore this observation, we examined the potential relationship of changes in all 10 proteins to the number of hits to the head that each fighter received. Only 1 of the biomarkers (UCHL1) demonstrated a significant regression (r^2^ = 0.7339, **Fig 4**). Notably, however, UCHL1 did not demonstrate a significant overall post- vs pre-effect (p = 0.934, median % change = 1.2), with approximately equal numbers of samples showing decreases and increases. The remaining proteins demonstrated r^2^ coefficients ranging from 0.005–0.09 (**S1 Fig**).

### miRNA biomarkers

**Fig 5:**
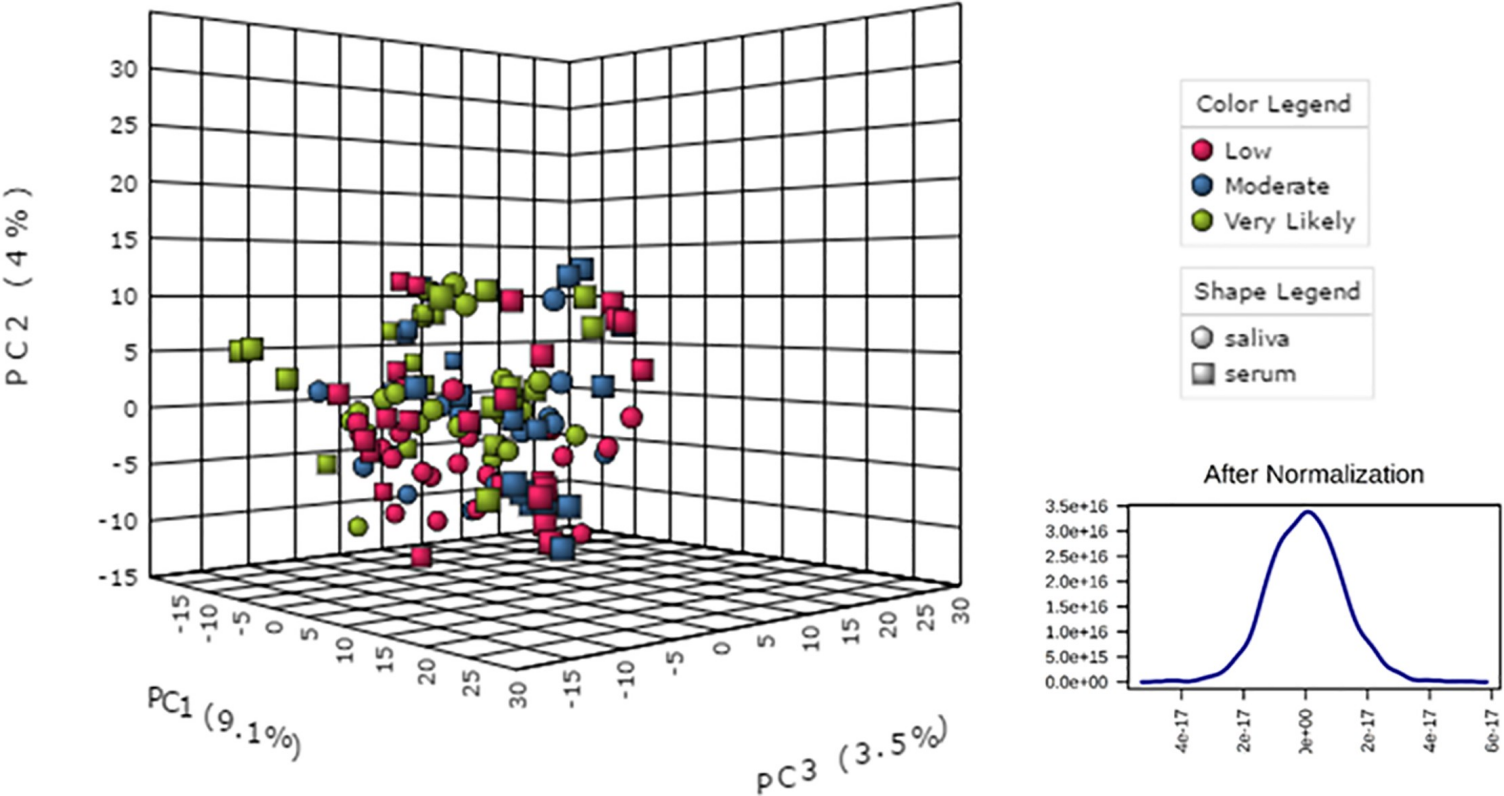
Principal component analysis (PCA) of miRNA data. The data were normalized across biofluid types and TBI likelihood prior to statistical analysis. The image at left shows intermixing of the samples, with only a slight suggestion of separation of Very Likely serum samples (green boxes) from the main data cloud. When all the data are collapsed, the change values are distributed in a highly normal fashion (lower right).

A total of 925 miRNAs were reliably quantified in the combined saliva and serum samples by RNA-Seq and subjected to downstream analysis. After normalization, the changes in miRNA values were visually screened for sphericity and normality prior to statistical analysis using principal component analysis (PCA) (**Fig 5**). The results demonstrated a generally unbiased data set regardless of the biofluid type, with no obvious outliers based on the clustering and the size of the PCA axes.

**Fig 6:**
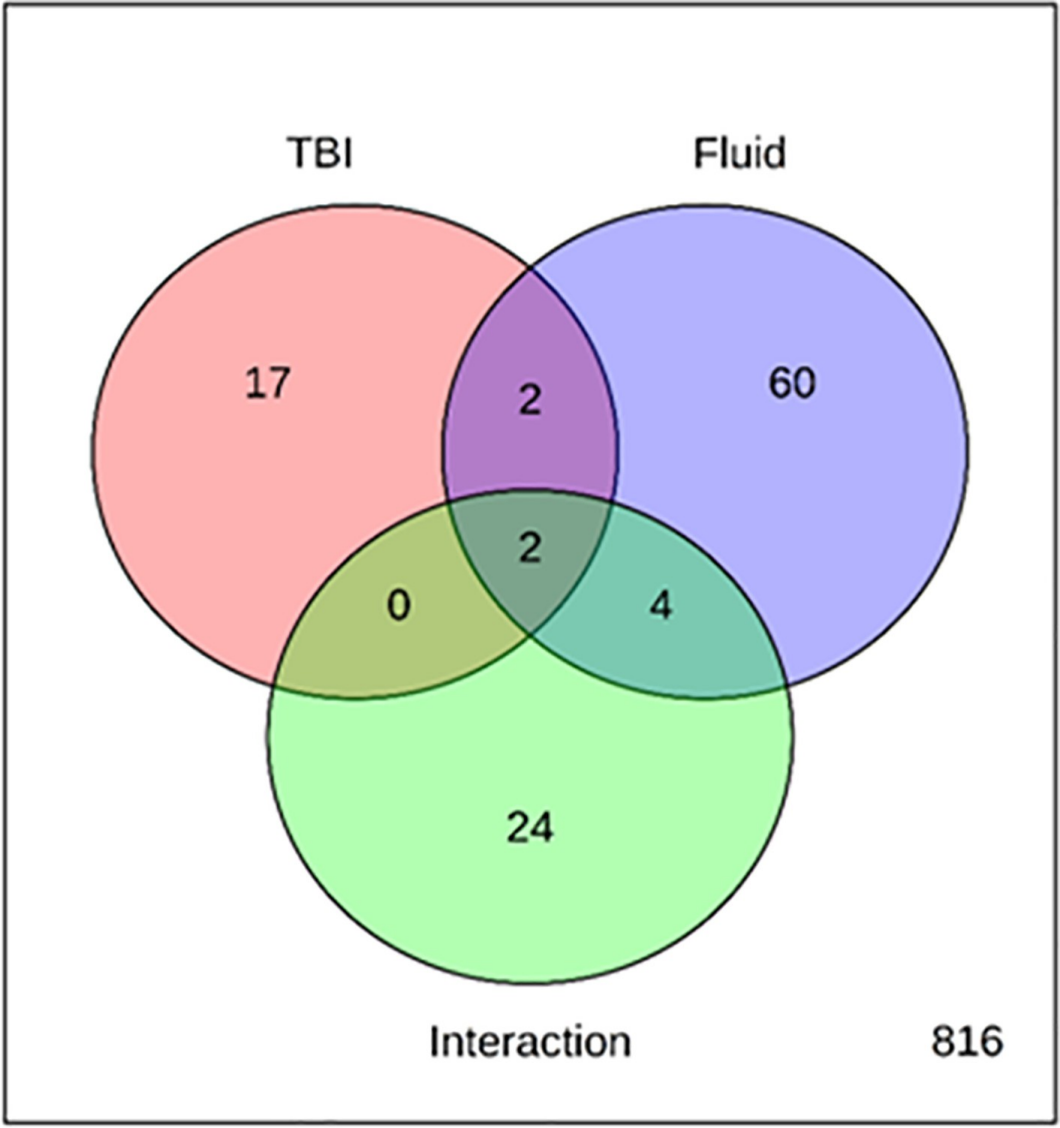
Effects of HTH (mTBI likelihood) on miRNA expression changes in serum and saliva. A total of 925 miRNAs were tested, with 21 showing a significant main effect of TBI likelihood, of which two also showed a significant main effect of Fluid and two showed a significant Fluid x TBI interaction.

After correcting for multiple testing (FDR < 0.15), a total of 21 miRNAs demonstrated significant changes according to the HTH/mTBI likelihood classification (**Fig 6, Table 5**). Of these, two also showed a significant effect of Fluid type and two showed an Interaction effect of Fluid type x TBI likelihood. To determine if the TBI-associated miRNAs might reflect release from central nervous system sources, we used the miRGator3.0 tool (http://mirgator.kobic.re.kr). A miRNA was considered “brain- enriched” if its median expression across multiple CNS sources exceeded the median expression in any of the 31 non-neural organs and 51 non-neural tissues in the miRGator 3.0 database. Of the 18 miRNAs with mapping information available, only two exhibited highest expression in the CNS, suggesting a possible CNS origin for the salivary or serum signature post-fight (**Table 5**). However, we also compared our list of miRNAs with those reported as detected in a previous study of CSF samples from TBI subjects [12] and noted that 16 of the 21 miRNAs were found at levels > 10 reads per million (RPM), 9 were found at > 100 RPM, 4 were found at > 1,000 RPM, and 3 were found at > 10,000 RPM (**Table 5**).

**Table 5:** miRNAs with changes related to TBI likelihood.

miRNA	TBI	Fluid	Interaction	Change Saliva	Change Serum	Top Human Tissues	Human CSF
miR-7-1-3p	0.147	0.853	0.417	↓	-	Fetal CNS, nose, pharynx, lymphoid, adipose	
miR-10a-5p	0.136	0.131	0.417	↓	↑	Kidney, stomach, uterus, liver, lung	++++
miR-10b-5p	0.119	0.234	0.739	↑	↑	Uterus, prostate, bladder, breast, head	++++
miR-20a-5p	0.136	0.987	0.396	↑	-	Kidney, lymphoid, uterus	+
**miR-30b-5p**	0.136	0.408	0.723	↑	↑	Breast, nose, testes, heart	++
**miR-92a-3p**	0.119	0.987	0.594	↓	↓	Kidney, fetal CNS, tonsil, adult CNS, lymphocyte	++++
**miR-122-5p**	0.119	0.024	0.162	-	↑	Germ line cells, liver, kidney	+++
miR-128-3p	0.147	0.850	0.803	↓	↑	CNS, lung, fetal lung, lymphoid	++
miR-155-5p	0.147	0.589	0.806	-	↑	Lymphoid, tonsil, umbilical cord	++
miR-455-5p	0.136	0.803	0.896	↓	↓	Skin, keratinocytes, uterus, heart, liver, cerebellum	+
miR-1307-3p	0.147	0.720	0.760	↓	↑	Pharynx, kidney, liver thyroid, head	++
miR-3146	0.119	0.649	0.844	↓	-	Lymphocytes, uterus, keratinocytes, kidney, tonsil	+
miR-3678-3p	0.147	0.922	0.821	↓	↓	Lymphocyte, Tonsil	
miR-376a-5p	0.021	0.535	0.749	↓	-	Skin, placenta, heart, CNS, testes	+
miR-4637	0.136	0.689	0.516	-	↑	Uterus, lymphoid, tonsil, keratinocytes, liver, CNS	
miR-4649-3p	0.119	0.091	0.139	↓	-	Nose, uterus, foreskin fibroblasts	
miR-4693-5p	0.119	0.320	0.812	-	↑	‘-	
miR-4766-5p	0.147	0.015	0.139	↓	-	Lymphoid, uterus, kidney, liver	+
miR-5694	0.147	0.649	0.665	↓	↓	Uterus, breast, brain, spleen, thymus	+
miR-6770-5p	0.136	0.235	0.825	↓	-	‘-	++
miR-6809-3p	0.119	0.269	0.668	↓	↓	‘-	+

Note: miRNAs in bold are displayed in subsequent figures. Top expressing tissues referenced from miRGator expression atlas of normal organs (http://mirgator.kobic.re.kr). Human cerebrospinal fluid (CSF) data referenced from Hicks and colleagues [12] based on reads per million (RPM) in control or TBI subject CSF according to log_10_ expression levels: + (>10 RPM), ++ (>100 RPM), +++ (>1,000 RPM), ++++ (>10,000 RPM).

**Fig 7:**
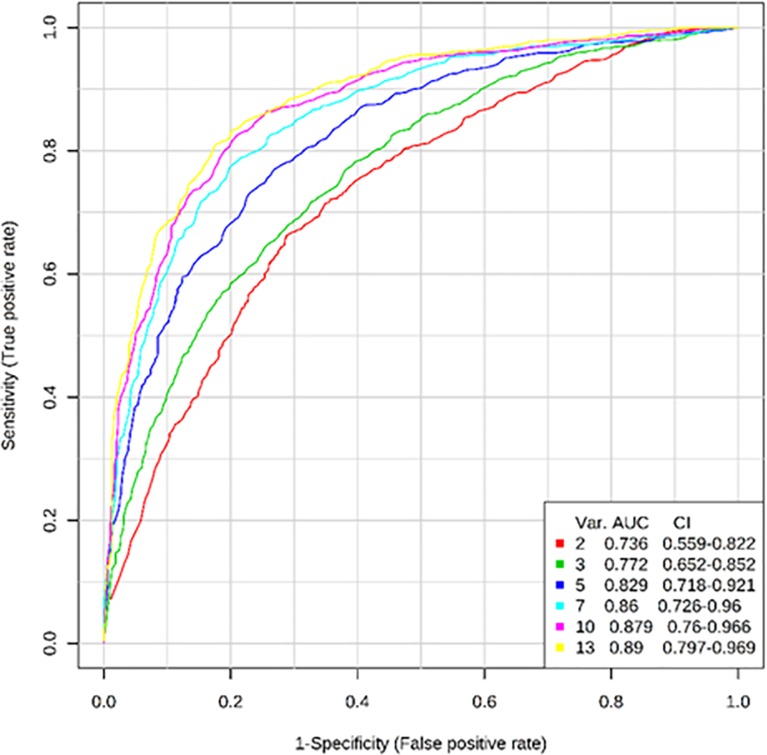
Accuracy of predicting highest and lowest TBI likelihood based on changes in miRNA expression from serum or saliva. Stepwise linear regression was used to pre-select an optimal number of miRNAs for prediction of Hits to the Head (HTH) values, and this set of 13 was subjected to 100-fold Monte Carlo Cross Validation (MCCV) using Random Forest, in order to estimate classification accuracy for distinguishing Very Likely from Low likelihood TBI samples.

Further examination of the miRNAs was performed in attempt to identify those with the strongest ability to predict the HTH level/mTBI likelihood, using Receiver Operating Curve (ROC) binary classification testing with feature selection and 100-fold Monte Carlo Cross Validation. In this case, we compared only the Low and the Very Likely TBI groups. In addition, we limited our selection of TBI predictors to those miRNAs that specifically showed a relationship between their expression changes and the number of hits to the head in the full set of samples (as determined by a stepwise linear regression). The results from this analysis yielded a multivariate prediction model with almost 90% accuracy (AUC = 0.89) for predicting mTBI in a given sample, regardless of fluid type, using as few as 13 miRNAs (**Fig 7**).

To further establish the validity of the miRNA biomarkers we identified, we also complemented the ROC analysis with a logistic regression based analysis that either combined or separated the two different biofluid sample types. The results indicated that the same 13 miRNAs could achieve perfect classification when *separate* logistic regression models (with different beta coefficients for each biofluid) were utilized (**Table 6**). Thus, we both serum and saliva appear to contain subsets of miRNAs that can accurately classify samples according to TBI likelihood, but that the information provided by each is somewhat distinct.

**Table 6:** Logistic regression model performance for binary HTH classification using 13 miRNAs.

Saliva Only Model			
	Predicted Low	Predicted Very Likely	% Accuracy
Observed Low	21	0	100
Observed Very Likely	0	21	100
			100
Serum Only Model			
	Predicted Low	Predicted Very Likely	% Accuracy
Observed Low	24	0	100
Observed Very Likely	0	24	100
			100
Combined Biofluid Model			
	Predicted Low	Predicted Very Likely	% Accuracy
Observed Low	38	7	84
Observed Very Likely	5	39	89
			87

**Fig 8:**
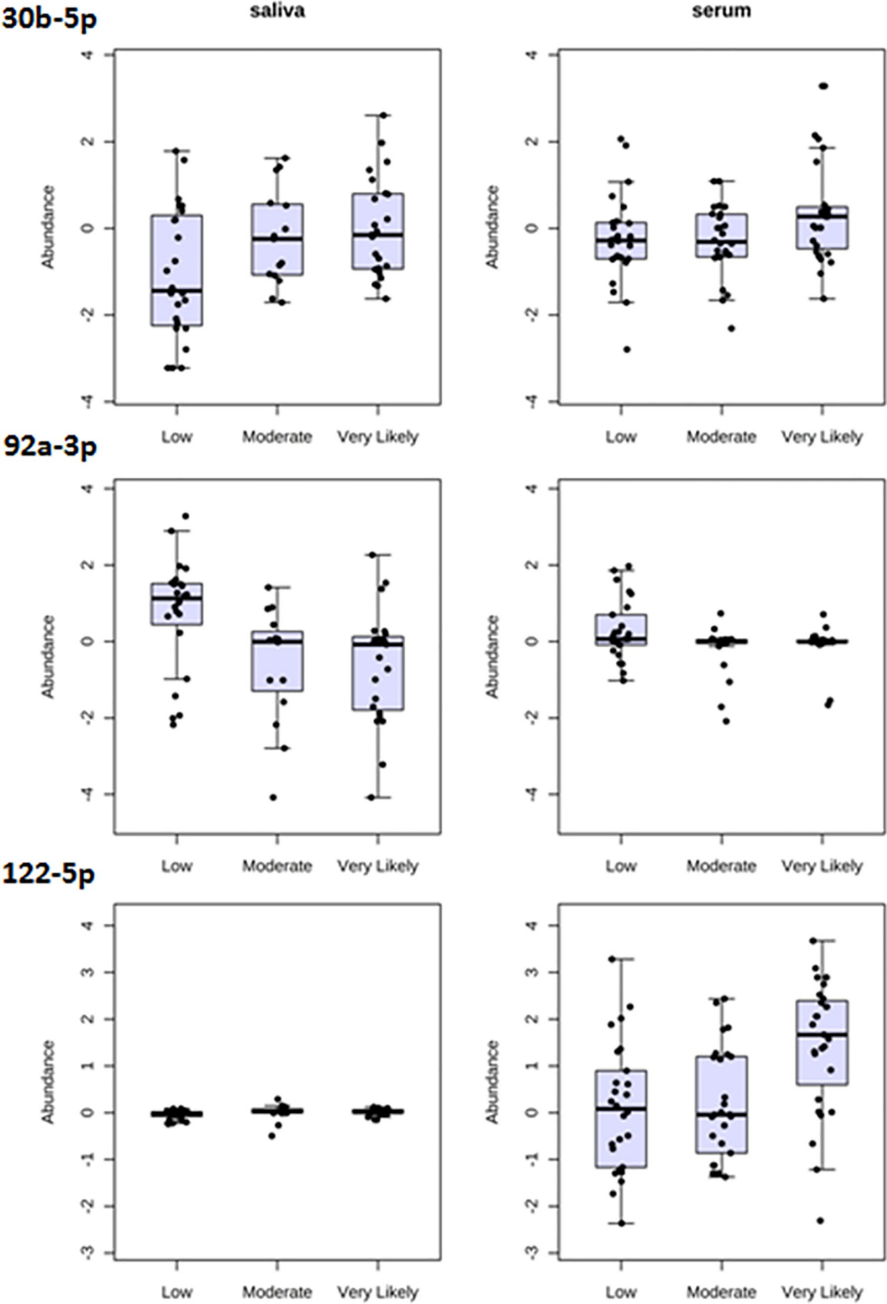
Changes in miRNA expression levels in saliva and serum post-fight, binned according to HTH levels /mTBI likelihood. Data displayed are whisker-box plots. Each row represents a different miRNA (three miRNAs are shown), and each dot represents the expression level of that miRNA in a sample. Note that some miRNAs showed a pattern of increase in both biofluids post-fight (30b-5p, top), while others showed a change that was most evident in only a single biofluid type (e.g., 92a-3p and 122-5p).

Examples of some of the 21 miRNAs in serum and saliva with significant changes in expression post-fight are shown in **Fig 8**. Interestingly, some of these miRNAs showed a pattern of increased expression in both biofluids post-fight (**Fig 8**, miR-30b-5p, top), while others showed a change that was most evident in only a single biofluid type. For example, miR-92a-3p was decreased largely in the saliva post-fight, while miR-122-5p was increased largely in the serum post-fight (**Fig 8**).

### Biological mapping of changed miRNAs

We further explored the biological relevance of the findings for the 21 significantly changed miRNAs using DIANA Tools miRpath v.3 (with FDR correction set <0.05). This analysis was based on predicted targets and indicated a distinct set of biological pathways was overrepresented in the target genes of the top miRNAs. The top 10 pathways defined within the Kyoto Encyclopedia of Genes and Genomes (KEGG) database were displayed along with the net expression change of each associated miRNA in comparisons of the highest and lowest HTH groups for each biofluid (**Table 7**). Notably, across all the most enriched pathways, the associated miRNAs displayed mixed effects, with several increasing and several decreasing. More than half of the miRNAs (n = 13) showed mixed directionality of changes in the two biofluids, with an increase or decrease in one biofluid accompanied by no change or a change in the opposite direction in the other biofluid. However, 7 miRNAs did show changes in the same direction in the two biofluids, including 2 that increased (miR-10b-5p, miR-30b-5p) and 5 that decreased (miR-3678-3p, miR-455-5p, miR-5694, miR-6809-3p, and miR-92a-3p).

**Table 7:** Biological pathways overrepresented by target genes of HTH-related miRNAs.

KEGG pathway	FDR	Genes	miRNAs	10a-5p	10b-5p	122-5p	128-3p	155-5p	20a-5p	30b-5p	3146	3678-3p	376a-5p	455-5p	4637	4649-3p	4693-5p	4766-5p	5694	6770-5p	6809-3p	7-1-3p	92a-3p
Proteoglycans in cancer	1.1E-06	102	20	-/+	+/+	/+	-/+	/+	+/	+/+	-/	-/-	-/	-/-	/+	-/	/+	-/	-/-	-/	-/-	-/	-/-
Mucin type O-Glycan biosynthesis	2.7E-05	16	12	-/+	+/+	/+	-/+	/+	+/	+/+	-/	n/a	n/a	n/a	n/a	-/	n/a	n/a	n/a	-/	-/-	-/	n/a
TGF-beta signaling pathway	2.7E-05	46	20	-/+	+/+	/+	-/+	/+	+/	+/+	-/	-/-	-/	-/-	/+	-/	/+	-/	-/-	-/	-/-	-/	-/-
FoxO signaling pathway	3.2E-05	75	17	-/+	+/+	/+	-/+	/+	+/	+/+	n/a	-/-	-/	n/a	n/a	-/	/+	-/	-/-	-/	-/-	-/	-/-
Ubiquitin mediated proteolysis	3.2E-05	80	19	-/+	+/+	/+	-/+	/+	+/	+/+	-/	-/-	n/a	-/-	/+	-/	/+	-/	-/-	-/	-/-	-/	-/-
Hippo signaling pathway	3.3E-05	76	16	-/+	+/+	n/a	-/+	/+	+/	+/+	-/	-/-	-/	n/a	n/a	-/	/+	-/	n/a	-/	-/-	-/	-/-
Axon guidance	5.9E-05	70	17	-/+	+/+	/+	-/+	/+	+/	+/+	n/a	-/-	-/	n/a	n/a	-/	/+	-/	-/-	-/	-/-	-/	-/-
Ras signaling pathway	0.0002	111	19	-/+	+/+	/+	-/+	/+	+/	+/+	-/	-/-	-/	n/a	/+	-/	/+	-/	-/-	-/	-/-	-/	-/-
AMPK signaling pathway	0.0002	67	20	-/+	+/+	/+	-/+	/+	+/	+/+	-/	-/-	-/	-/-	/+	-/	/+	-/	-/-	-/	-/-	-/	-/-
Glutamatergic synapse	0.0003	61	17	-/+	+/+	/+	-/+	/+	+/	+/+	-/	-/-	-/	n/a	n/a	-/	/+	-/	n/a	-/	-/-	-/	-/-

+/- symbols indicate direction of change for saliva (left) and serum (right) samples in Very Likely TBI vs Low probability TBI groups, respectively (minimum change +/- 0.1). Absence of one symbol indicates no change. n/a indicates a miRNA not in that group.

Notably, of the top ten ranked KEGG pathways, four were of particular interest for their potential relevance to TBI. These pathways included Ubiquitin-mediated proteolysis, Transforming growth factor-beta (TGF-beta), Axon guidance, and Glutamatergic synapse. Within each of these pathways a total of 46–80 genes were targeted by a total of 20 of the miRNAs. We examined these findings further using DIANA Tools to display maps of each pathway with the genes targeted by 1 or more miRNAs (**S2–S5 Figs**).

### Correlation of miRNA changes and functional changes

Finally, we examined the quantitative relationships of the 21 most significantly changed miRNAs from the two-way ANOVA and the top-changed functional measures as well as actual HTH values, across all subjects. This analysis revealed a single nominally significant negative correlation between the changes in serum miR-4766-5p levels and TLEC functional measures (**Table 8**). Notably, this same miRNA also had a weak positive correlation between its changes in the serum and the balance score differences in the DSB_Bal test. In contrast to these nominally significant correlations with functional outcomes, we observed several highly significant correlations with the HTH values themselves that survived Bonferroni correction (n = 7 in salivary miRNAs, n = 3 serum miRNAs, and n = 8 in the combined samples).

**Table 8:** Correlations between changes in miRNA levels (post-fight), HTH, and functional measures.

		Hits to the Head			Two Legs Eyes Closed Balance			Digit Span Backwards Balance		
Change	miRNA	All	Saliva	Serum	All	Saliva	Serum	All	Saliva	Serum
-/+	miR-10a-5p	0.013	0.149	0.031	-0.146	-0.029	-0.206	-0.006	-0.002	0.036
+/+	miR-10b-5p	**-0.583**	0.273	**-0.610**	-0.147	0.078	-0.228	0.202	0.294	0.012
/+	miR-122-5p	**0.372**	0.336	**0.386**	-0.192	-0.046	-0.278	0.034	-0.066	0.086
-/+	miR-128-3p	**0.280**	0.355	0.268	0.040	0.076	0.026	0.079	-0.011	0.157
-/+	miR-1307-3p	0.237	**0.474**	0.185	-0.102	-0.018	-0.145	-0.061	0.000	-0.070
-/+	miR-155-5p	0.079	0.107	0.099	-0.016	0.174	-0.039	0.159	0.281	0.231
+/	miR-20a-5p	-0.136	0.096	-0.175	-0.096	-0.168	-0.058	-0.025	-0.030	0.038
+/+	miR-30b-5p	0.070	0.197	-0.028	-0.006	-0.117	0.078	0.216	0.097	0.359
-/	miR-3146	0.124	0.251	-0.325	-0.181	-0.185	-0.182	-0.221	-0.274	-0.069
-/-	miR-3678-3p	**0.421**	**0.658**	-0.096	0.095	0.120	0.091	0.007	0.049	0.004
-/	miR-376a-5p	**0.444**	**0.574**	0.210	-0.025	-0.037	0.020	-0.171	-0.124	-0.278
-/-	miR-455-5p	0.254	0.36	0.118	-0.189	-0.187	-0.215	-0.195	-0.211	-0.176
/+	miR-4637	-0.210	0.023	-0.250	0.089	-0.009	0.159	-0.019	-0.253	0.298
-/	miR-4649-3p	0.058	0.055	-0.019	0.001	-0.002	-0.005	-0.103	-0.098	-0.184
/+	miR-4693-5p	-0.006	-0.031	-0.008	0.115	0.090	0.164	-0.015	-0.088	0.324
-/	miR-4766-5p	0.060	**0.488**	0.043	-0.063	-0.045	**-0.385**	-0.098	-0.121	0.324
-/-	miR-5694	0.055	-0.258	0.094	-0.058	0.027	-0.180	-0.037	0.067	-0.204
-/	miR-6770-5p	**0.455**	**0.524**	**0.387**	0.104	0.078	0.156	0.141	0.130	0.202
-/-	miR-6809-3p	**0.293**	**0.439**	0.079	-0.102	0.095	-0.132	-0.062	0.074	-0.213
-/	miR-7-1-3p	0.017	0.287	-0.049	-0.006	0.107	-0.075	-0.055	-0.128	0.041
-/-	miR-92a-3p	**0.300**	**0.412**	-0.013	-0.105	-0.184	-0.001	-0.122	-0.151	0.005

Pearson correlations between HTH values and changes in miRNa levels were adjusted using Bonferroni FDR < 0.05 (bold)

Correlations between TLEC, DSB_Bal and changes in miRNA levels were interpreted without FDR correction (p<0.05)

### Temporal analysis of miRNA changes

**Fig 9:**
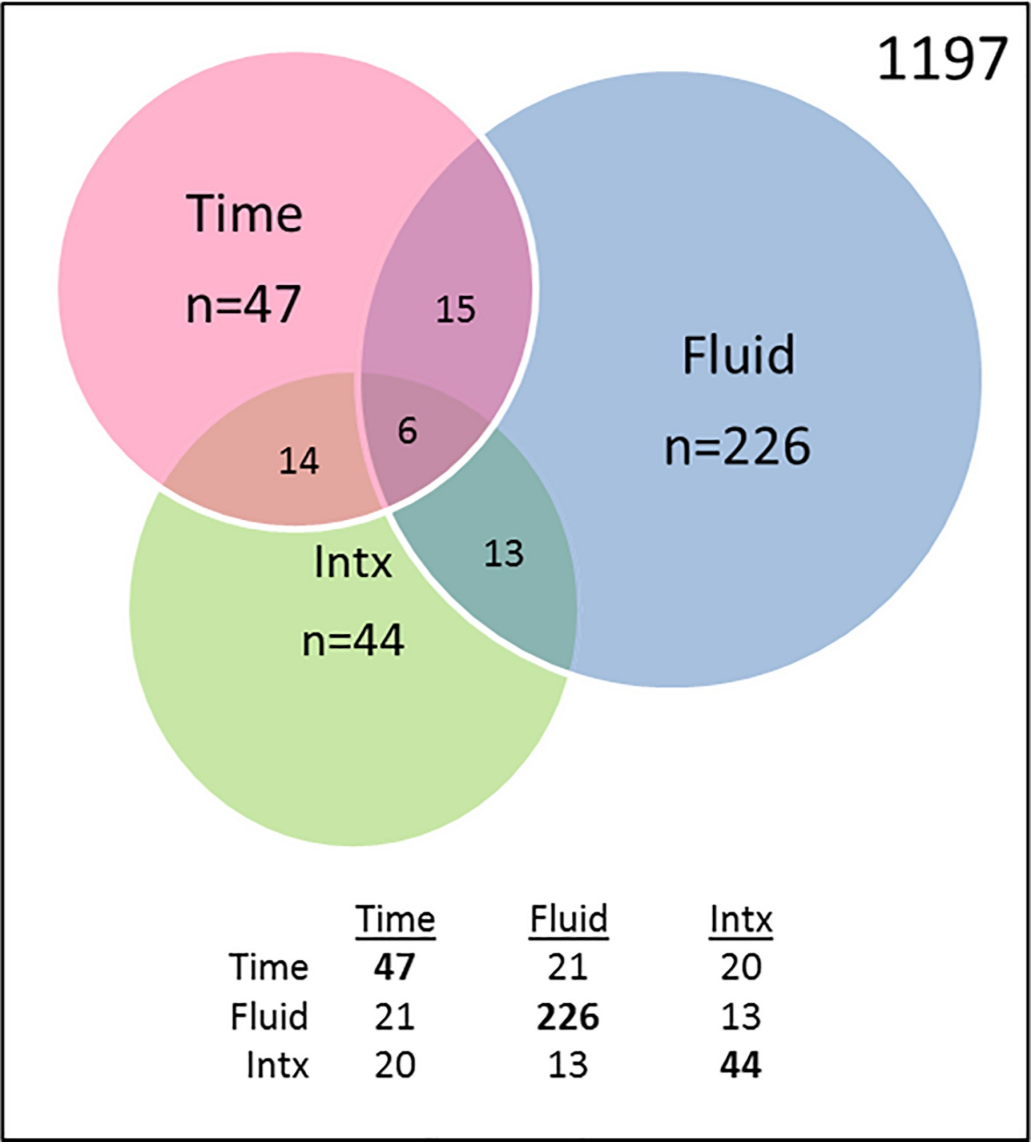
miRNAs with changes in abundance due to Time, Fluid, and Interaction effects in serum and saliva. Venn diagram denotes the number of genes with significant effects of Time, Fluid, or the Interaction (Intx) of Time x Fluid.

In addition to probing for changes in expression based solely on HTH values/TBI likelihood, we also sought to identify miRNAs with more complex and potentially more biologically relevant changes in expression that might have escaped detection when combined across all the sampling times. This was accomplished through a General Linear Model encompassing Time and Sample Type. Using this approach, out of 1197 tested miRNAs, we found 47 miRNAs with significant effects of Time, 226 with significant effects of Fluid and 44 with significant effects of the Interaction between Time and Fluid (**Fig 9**). Since our goal was to identify temporal effects that might reflect the occurrence of an mTBI event in either biofluid, we focused exclusively on the 47 miRNAs with significant effects of Time (**Table 9**). Of these, 21 had significant effects of Fluid, and 20 had significant Interaction effects, indicating that their changes showed different temporal effects in the two biofluids. From the 47, we identified 25 with fairly distinct patterns by visual inspection following supervised clustering analysis (**S1 Table**).

**Table 9:** 47 miRNAs with significant effect of time in relation to MMA fight in saliva and serum.

miRNA	Time (47)	Fluid (21)	Interaction (20)	Pattern	Top Human Tissues	Human CSF
miR-4529-3p	0.001048*	0.000171*	0.000260*	Delayed Serum	CNS	
miR-4782-5p	0.001478*	0.771777	0.007645*		PBMC, Tonsils	
miR-4495	0.002438*	0.001105*	0.068731		Breast, Umbilicus	
miR-3663-3p	0.004628*	0.393426	0.006147*		CNS	
miR-203a-3p	0.005004*	0.953766	0.019048*		Skin, Head/Limb	++++
miR-3170	0.005494*	0.082871	0.001233*	Acute Saliva	Liver, Kidney	+
miR-5588-5p	0.005613*	0.000210*	0.342059	Delayed Serum	Liver, Lymphocyte	+
miR-3677-5p	0.005844*	0.000047*	0.277949		Neurospheres	
miR-4485-3p	0.006945*	0.002592*	0.006234*		Germ cell, Tonsil, Nose	++
miR-6755-5p	0.007367*	0.429112	0.008562*		-	
miR-6855-3p	0.010420*	0.15248	0.013031*		-	
miR-8089	0.013930*	0.157337	0.960979	Delayed Serum	-	++
miR-365a-5p	0.014130*	0.012816*	0.125236		Lymphocyte, Pigmented cell	
miR-550a-3-5p	0.014394*	0.000366*	0.014623*	Delayed Serum	Nose, Adipose Tissue	
miR-3919	0.015643*	0.000245*	0.475008	Acute Saliva	CNS	
miR-499a-5p	0.016956*	0.184234	0.529812		Heart, Kidney, Germ cell	+
miR-433-3p	0.017808*	0.000472*	0.535641	Acute Saliva	Pharynx, CNS	+
miR-139-5p	0.019453*	0.000483*	0.016949*	Delayed Serum	Bladder, Kidney, Spleen	+
miR-8082	0.021022*	0.013965*	0.027255*		-	++
miR-2682-5p	0.021615*	0.000003*	0.411552	Acute Saliva	CNS	+
miR-548ab	0.021980*	0.891496	0.018717*		Lymphocyte, Tonsil, CNS	
**miR-3678-3p**	0.022890*	0.002552*	0.24893	Delayed Serum	Lymphocyte, Tonsil	
miR-4632-3p	0.024974*	0.190454	0.020774*	Acute Saliva	Spleen	
miR-5583-5p	0.025676*	0.012704*	0.399673		Embryonic kidney	+
miR-6870-3p	0.026225*	0.028773*	0.109315	Acute Saliva	-	
miR-1270	0.026246*	0.009370*	0.361532	Delayed Serum	Lymphocyte, Tonsil, Thyroid	+
miR-3664-3p	0.027180*	0.102718	0.023126*	Delayed Serum	Liver, Tonsil	
miR-421	0.028354*	0.055815	0.014727*	Delayed Serum	Stem cell, Kidney	++
let-7b-3p	0.028535*	0.070946	0.839897	Acute Saliva	Umbilicus, Nose	
miR-4800-5p	0.029069*	0.942453	0.412773		Lymphocyte, Tonsil, Lung	++
miR-4749-5p	0.029116*	0.378594	0.885014		Lymphocyte, Tonsil	
miR-30c-1-3p	0.029679*	0.529053	0.216003	Delayed Serum	Heart, Nose	++
miR-616-5p	0.029836*	0.41128	0.177306		Nose, Adipose tissue	
miR-135b-5p	0.031594*	0.422428	0.031404*		Nose, Testes	
miR-6840-5p	0.037916*	0.264125	0.274613		-	+
miR-608	0.038108*	0.003982*	0.532572	Acute Saliva	Breast, Spleen, Thymus	+
miR-374c-5p	0.038280*	0.209441	0.412421		CNS	+
miR-4760-5p	0.040453*	0.275308	0.027557*	Acute Saliva	Keratinocytes, CNS	+
miR-4727-3p	0.042900*	0.045677*	0.189207	Delayed Serum	Stem Cell, Vertebral disc	
miR-501-3p	0.043792*	0.113446	0.042896*	Delayed Serum	Nose, Adipose tissue	+
miR-3187-5p	0.043874*	0.579419	0.189533		PBMC, Tonsil	
miR-3118	0.046986*	0.134052	0.028899*	Acute Saliva	PBMC, Tonsil Plasma Cell	+
miR-766-3p	0.047390*	0.212496	0.78748		Pharynx, Tonsil, Nose	+
**miR-6809-3p**	0.047799*	0.000051*	0.411403	Delayed Serum	-	+
miR-601	0.049388*	0.056646	0.113978	Acute Saliva	Placenta, Cerebellar Cortex	+
miR-4660	0.049499*	0.012181*	0.210414	Acute Saliva	Pigment cell, Tonsil	
miR-4699-5p	0.049827*	0.000083*	0.031381*		Adipose tissue, Nose, Liver	++

**Fig 10:**
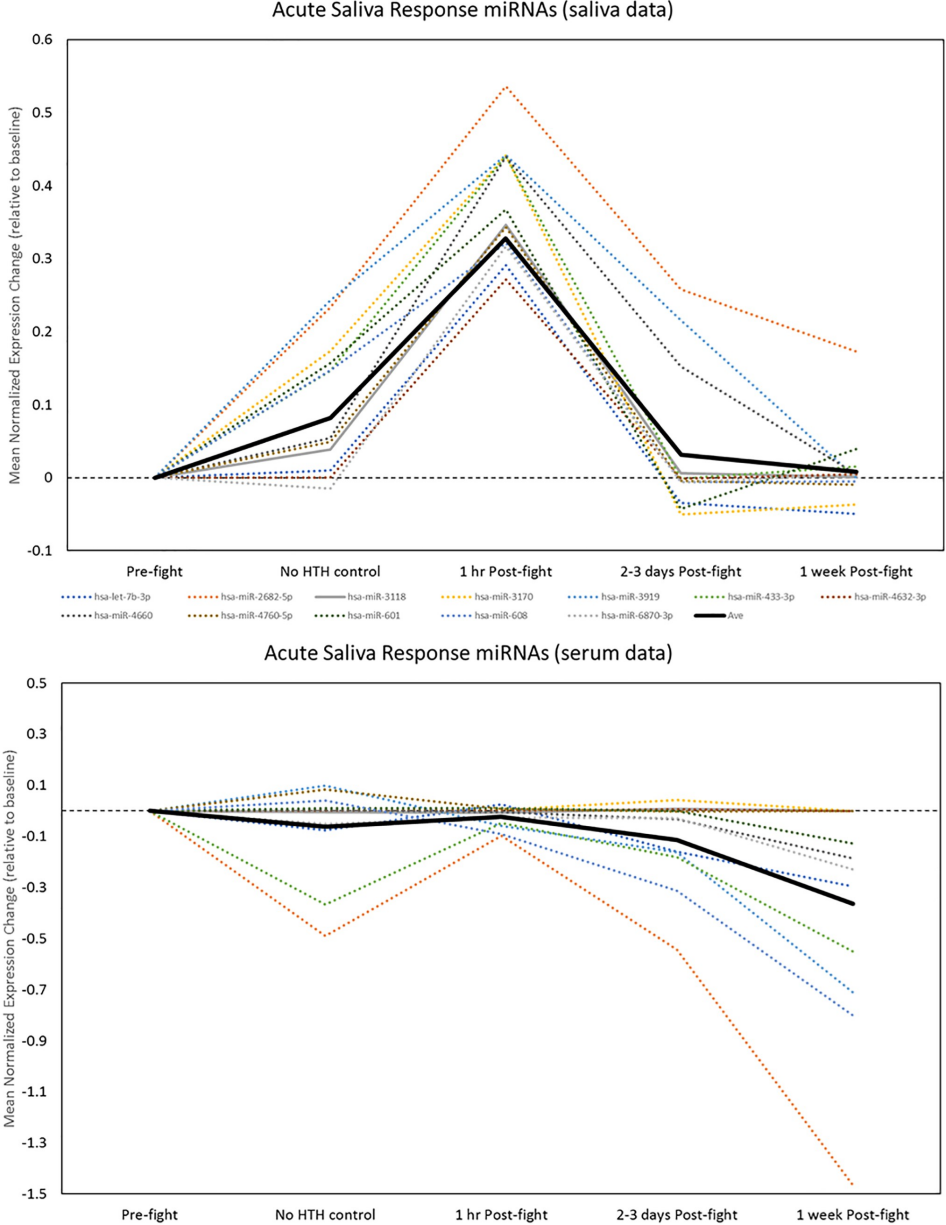
Detection of acute saliva response (ASR) miRNAs. 12 miRNAs were identified with robust temporal effects (all increases) at the 1 hr Post-fight time point (blue shaded area) in saliva samples (upper) that exceeded those at the non-specific exercise- or event-related timepoint (green shaded area). Note that most of the miRNAs returned to near baseline by 2–3 days Post-fight. The pattern for the same miRNAs was distinctly different in serum (several were unchanged and several had delayed decreases).

Asterisks indicate change according to Time, Fluid or the interaction. Bold miRNAs were also changed due to HTH levels. Pattern column indicates miRNAs shown in Figs 9 & 10. Other conventions same as Table 5.

Visual inspection of the temporal patterns of significant changed miRNAs was used to identify potential biomarkers with salient patterns of either acute, delayed or sustained effects at the post-fight timepoints of interest. To be considered a specific HTH-related pattern, the magnitude of the observed change had to exceed the magnitude of non-specific changes seen on the day of the fight associated with the event and possibly exertion, but not hits to the head (HTH). We used two criteria for this procedure: the post-fight change (at any time point) had to exceed 1.3-fold (a log2 change of +/- 0.28), and it had to exceed the magnitude of change in the HTH–control group by at least two-fold. These two simple criteria revealed two sets of miRNAs with highly-distinct patterns in the biofluid samples. The first set of miRNAs showed an acute increase in saliva immediately post-fight that then returned to normal levels on days 2–3 and 1 week post-fight. This pattern was evident primarily in saliva samples and accurately described 12 of the 47 miRNAs with significant ANOVA effects (**Fig 10, upper; S1 Table**). We termed these Acute Saliva Response (ASR) miRNAs. Remarkably, these same miRNAs demonstrated a distinctly different pattern of change in the serum samples. Specifically, none were increased, a small number showed no change, and several showed a delayed decrease, beginning at 2–3 days post-fight (**Fig 10, lower**).

**Fig 11:**
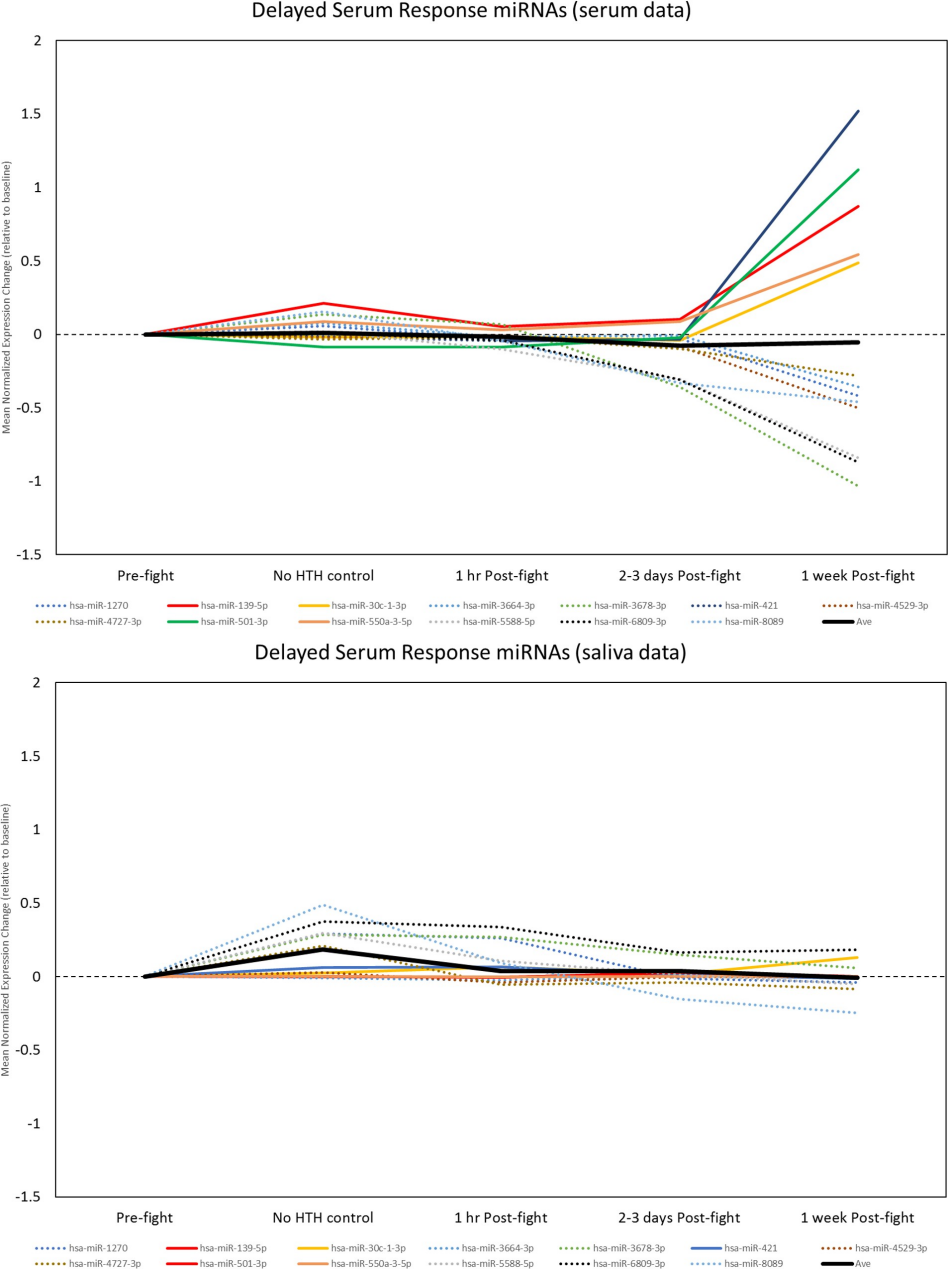
Detection of miRNAs with delayed serum response (DSR). A subset of miRNAs were identified with predominantly delayed increases (solid lines) and decreases (dashed lines) in serum at 1 week Post-fight (upper, blue shaded area) that exceeded those at the non-specific exercise- or event-related timepoint (green shaded area). Note that these miRNAs were unchanged or showed some evidence for non-specific increases in saliva (lower).

The second pattern was a delayed effect, usually a graded increase or decrease in expression on days 2–3 that reached a peak at 1 week post-fight, and was not present at the initial post-fight time point. This pattern was highly apparent in serum samples, and accurately described changes in 13 of the 47 miRNAs (**Fig 11, upper; S1 Table**). We termed these Delayed Serum Response (DSR) miRNAs. Notably, these same miRNAs did not exhibit this pattern in the saliva samples. Rather, most were either unchanged or showed a trend for modestly increased expression at earlier time points, including potentially non-specific or exercise-related changes (**Fig 11, lower**).

To ascertain the potential for the saliva and serum miRNAs to reflect release from central nervous system sources, we used the miRGator3.0 tool. A miRNA was considered “brain- enriched” if its median expression across multiple CNS sources exceeded the median expression in any of the 31 non-neural organs and 51 non-neural tissues in the miRGator 3.0 database. Of the 11 ASR miRNAs with mapping information available, four were identified as brain-enriched, suggesting possible CNS origin for the salivary miRNAs that increased within an hour post-fight (**Table 9**). This finding stands in contrast with the DSR miRNAs, where of the 11 serum miRNAs with mapping information available, only 1 was found to be brain-enriched (**Table 9**).

### Biological mapping of miRNAs with HTH-related acute or delayed changes

We further explored the biological relevance of the findings for the 12 miRNAs with notable increases in the saliva at the acute 1 hour post-fight time point and the 13 miRNAs identified in the serum with delayed changes (both increases and decreases) that peaked at 1 week post-fight. This analysis was performed using DIANA Tools miRpath 3.0, with the top 15 KEGG pathway enrichments identified for each set of miRNAs. Among the pathways enriched in the predicted targets of the acute saliva response miRNAs were several related to brain function, including Prion disease, Long-term depression, Glutamatergic synapse, Axon guidance, Amphetamine addiction, and Cocaine addiction (**Table 10**). Because these miRNAs were all increased (denoted by red upward arrows), the implication is that each of these brain-related pathways (and the others listed) were potentially being suppressed.

**Table 10:** Top biological pathways overrepresented by acute saliva response miRNAs.

KEGG pathway	FDR	Genes	miRNAs	let-7b-3p	2682-5p	3118	3170	3919	433-3p	4632-3p	4660	4760-5p	601	608	6870-3p
Prion diseases	7.0E-11	7	5	+	+	n/a	n/a	n/a	+	n/a	n/a	+	+	n/a	n/a
**Long-term depression**	3.4E-06	28	10	+	+	+	+	+	+	+	+	+	n/a	+	n/a
Hippo signaling pathway	7.1E-06	46	11	+	+	+	+	+	+	+	+	+	+	+	n/a
Proteoglycans in cancer	1.2E-05	60	11	+	+	+	+	+	+	n/a	+	+	+	+	+
Signaling pathways regulating pluripotency of stem cells	1.5E-05	51	11	+	+	+	+	+	+	n/a	+	+	+	+	+
**Thyroid hormome signaling pathway**	1.8E-05	41	11	+	+	+	+	+	+	n/a	+	+	+	+	+
N-Glycan biosynthesis	1.0E-04	15	8	+	+	+	+	+	+	n/a	+	n/a	n/a	+	+
**Glutamatergic synapse**	0.0008	36	11	+	+	+	+	+	+	n/a	+	+	+	+	+
Glycosaminoglycan biosynthesis—heparan sulfate/herapan	0.0009	10	8	n/a	+	+	n/a	+	n/a	n/a	+	+	+	+	+
**Axon guidance**	0.0019	43	10	+	+	+	+	+	+	n/a	+	+	+	+	n/a
Adherens junction	0.0019	29	6	+	+	n/a	n/a	+	+	n/a	+	+	n/a	n/a	n/a
Amphetamin addiction	0.0019	21	10	+	+	+	+	+	+	n/a	+	+	+	+	n/a
Estrogen signaling pathway	0.0019	31	11	+	+	+	+	+	+	n/a	+	+	+	+	+
Cocaine addiction	0.0035	18	10	+	+	+	+	+	+	n/a	+	+	+	+	n/a
**ErbB signaling pathway**	0.0036	30	9	+	+	+	n/a	+	+	n/a	+	+	+	+	n/a

Note: Conventions same as Table 8. Pathways in bold were the same or highly-related to pathways enriched in the delayed serum response miRNA targets.

Several KEGG pathways related to brain function were also among those enriched in the predicted targets of the delayed serum response miRNAs, including Axon guidance, Long-term potentiation, and Glutamatergic synapse (**Table 11**). Because some of these miRNAs were increased and others decreased (red arrows and green arrows, respectively), it is more difficult to interpret the consequences of these findings. Notably, several of the pathways enriched with miRNA targets in Table 10 and Table 11 were the same or highly-related to each other (e.g., Long-term depression and Long-term potentiation). These similar enrichment findings were further examined at the gene level within selected pathways.

**Table 11:** Top biological pathways overrepresented by delayed serum response miRNAs.

KEGG pathway	FDR	Genes	miRNAs	miR-1270	miR-139-5p	miR-30c-1-3p	miR-3664-3p	miR-3678-3p	miR-421	miR-4529-3p	miR-4727-3p	miR-501-3p	miR-550a-3-5p	miR-5588-5p	miR-6870-3p	miR-8089
Mucin type-O-Glycan biosynthesis	2.9E-07	11	6	n/a	n/a	n/a	-	n/a	+	n/a	n/a	n/a	+	-	-	-
Adrenergic signaling in cardiomyocytes	2.3E-05	48	12	-	+	+	-	-	+	-	n/a	+	+	-	-	-
ErbB signaling pathway	0.0002	30	12	-	+	+	-	-	+	-	n/a	+	+	-	-	-
ECM-receptor interaction	0.0004	20	8	-	n/a	n/a	-	-	+	n/a	n/a	+	+	n/a	-	-
Lysine degradation	0.0004	16	10	-	n/a	+	-	-	+	-	n/a	+	n/a	-	-	-
Axon guidance	0.0004	43	12	-	+	+	-	-	+	n/a	-	+	+	-	-	-
Proteoglycans in cancer	0.0015	65	13	-	+	+	-	-	+	-	-	+	+	-	-	-
Estrogen signaling pathway	0.0029	33	12	-	+	+	-	-	+	-	n/a	+	+	-	-	-
Glioma	0.0047	22	11		+	+	-	-	+	-	n/a	+	+	-	-	-
Thyroid hormone synthesis	0.0049	20	8	-	n/a	n/a	-	-	+	-	n/a	+	+	n/a	-	-
Oxytocin signaling pathway	0.0077	51	13	-	+	+	-	-	+	-	-	+	+	-	-	-
TGF-beta signaling pathway	0.0085	25	11	-	+	+	-	-	+	-	n/a	+	+	n/a	-	-
Long-term potentiation	0.0085	26	12	-	n/a	+	-	-	+	-	-	+	+	-	-	-
Glutamtergic synapse	0.0125	33	10	-	n/a	+	-	-	+	-	n/a	+	+	n/a	-	-
Prostate cancer	0.0165	30	11		+	+	-	-	+	-	n/a	+	+	-	-	-

Note: Conventions same as Table 8. Pathways in bold were the same or highly-related to pathways enriched in the acute saliva response miRNA targets.

The first pathway that we directly compared was the Glutamatergic synapse pathway (**S6 Fig**). We noted that many of the same genes were targeted by miRNAs found in saliva or serum. Some exceptions to the overlapping targets included SLC1A2/EAAT2 (only targeted by acute response salivary miRNAs) and Glutaminase/GLS2 and the vesicular glutamate transporter/SLC17A7 (only targeted by the delayed response serum miRNAs).

Possibly related to the Glutamatergic synapse pathway findings, we also found evidence of potentially paradoxical actions of salivary and serum derived miRNAs on two brain-related pathways involved in learning and memory–Long-term depression (LTD; targeted by acute response salivary miRNAs) and Long-term potentiation (LTP; targeted by delayed response serum miRNAs) (**S7 Fig**). These two biological processes are critical for the process of synaptic plasticity, with LTP promoting the insertion of post-synaptic glutamate (AMPA) receptors and enhancing synaptic growth, while LTD functions to internalize AMPA receptors and reduce post-synaptic responses.

### Combined analysis of temporal patterns in functional and miRNA data

#### Saliva data

**Fig 12:**
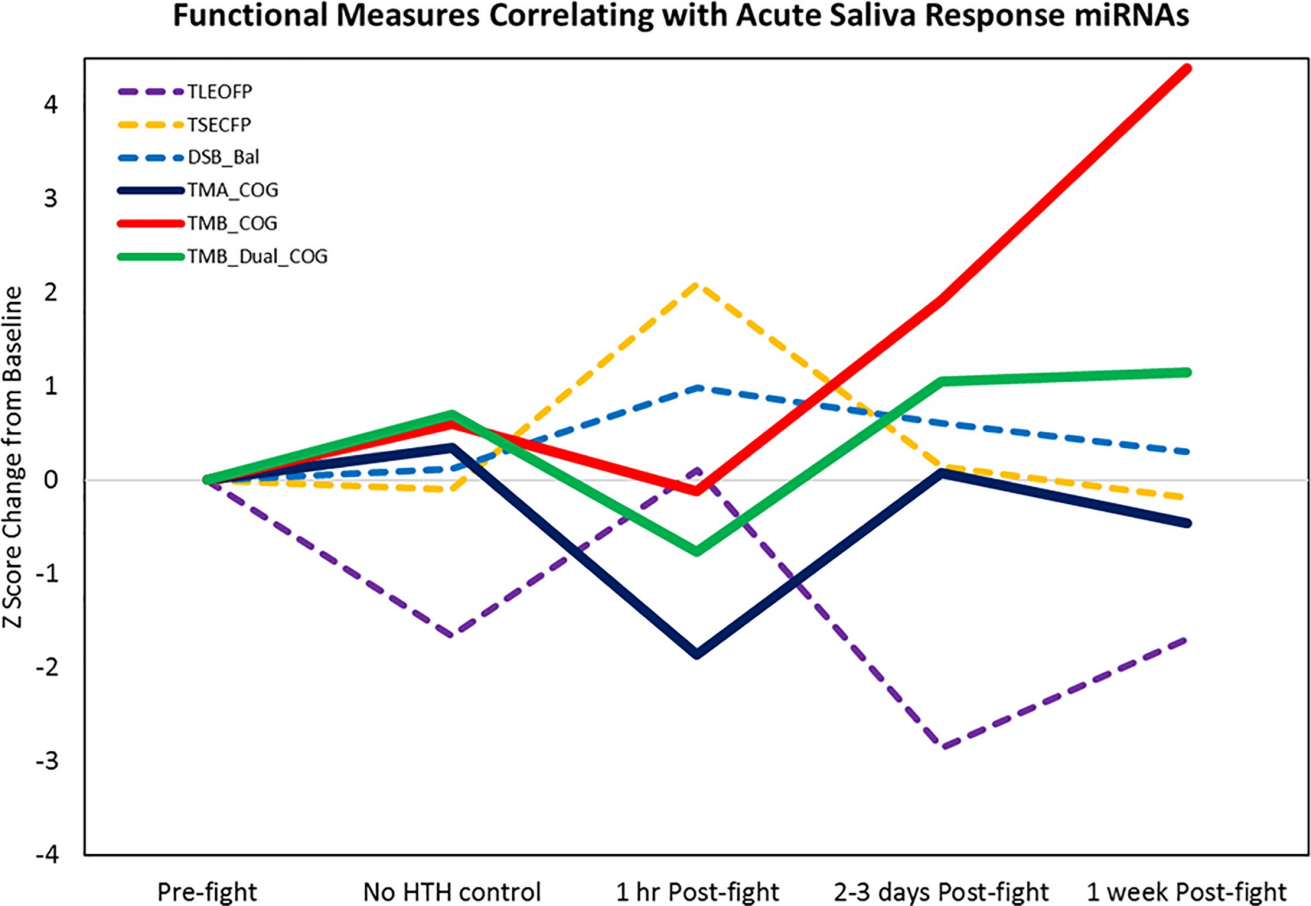
Functional measures correlated with acute saliva response miRNAs. Solid lines show cognitive measures (higher values indicate better performance). Dashed lines show normalized body sway measures (higher values indicate worse performance). Note that cognitive measures showed a trend for drop in performance at the 1 hr post-fight time point, while body sway showed an increase at the same time point. Also note that two of the cognitive measures (TMB_COG and TMB_Dual_COG) showed an apparent learning effect (improved performance across time, other than the immediate post-fight time point). A learning effect was also seen in 1 of the balance measures (TLEOFP), with decreased body sway evidence across time, other than the immediate post-fight time point.

Because we were able to identify temporal changes in the saliva and serum miRNA data, we also examined the balance and cognitive score data to detect those which might show the largest changes at particular timepoints and correlate with the ASR or DSR miRNAs. This was first performed using PCA on a total of 12 ASR miRNAs and 14 functional measures in 39 post-fight saliva samples with functional data. Our results indicated that 3 factors described approximately half the variance in the combined data. Factor 1 was the maximal loading component of 9/12 miRNAs and 4 functional measures (**S2 Table**), although some miRNAs and functional measures loaded strongly on multiple components. Notably, most Factor 1 loading saliva miRNAs showed large positive weights, along with several functional measures indicating increased body sway. In contrast, only 1 saliva miRNA showed a large negative weight on Factor 1, along with multiple functional measures indicating decreased cognitive performance (TMA_COG, TMB_Dual_COG, and TMB_COG). Graphical display of these data revealed a likely learning effect in one of the balance measures (TLEOFP), with decreased body sway evidence across time, other than the immediate post-fight time point (**Fig 12**).

#### Serum data

**Fig 13:**
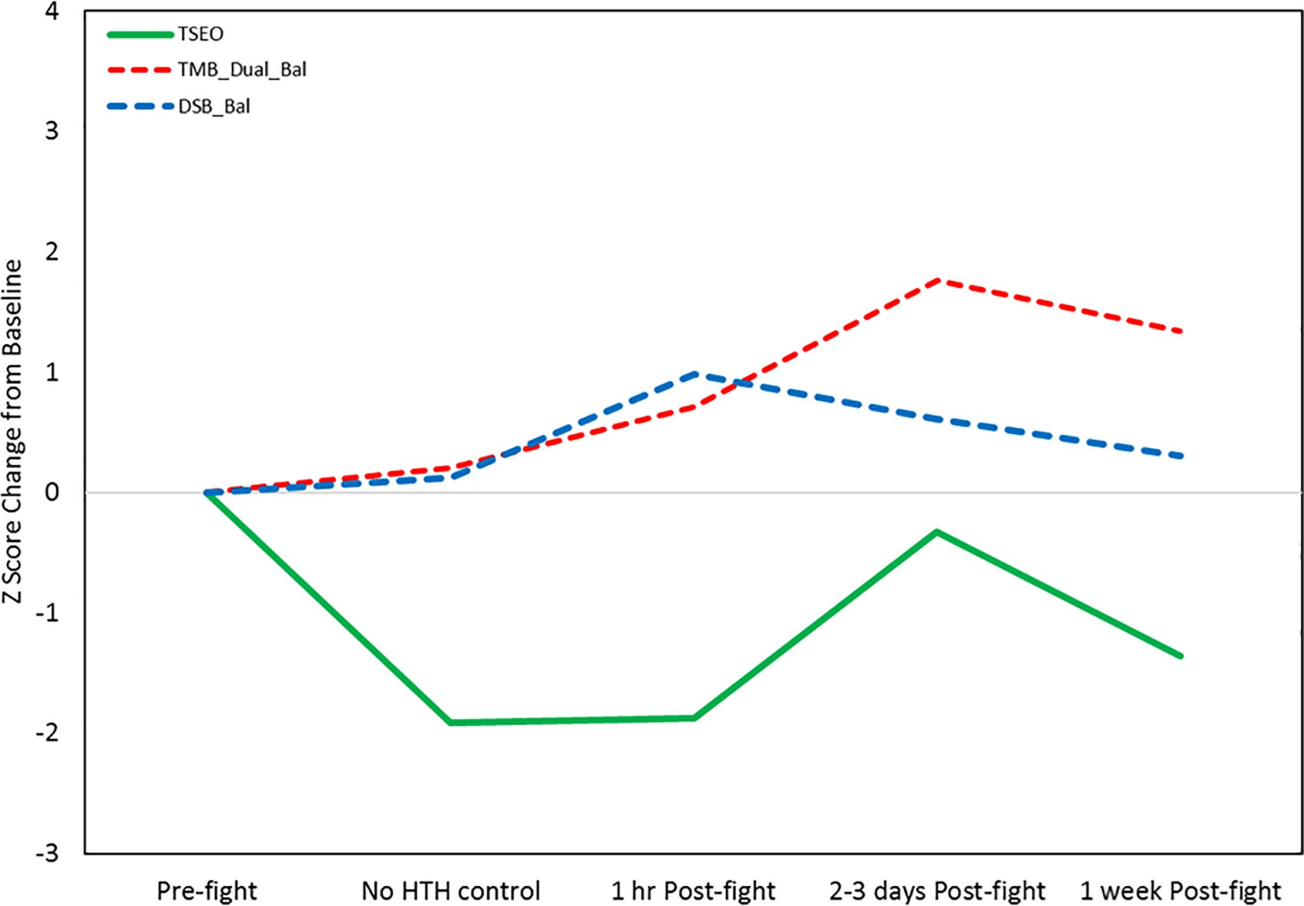
Functional measures correlated with delayed serum response miRNAs. Solid line shows a balance measure (TSEO) with apparent learning effects (decreased sway at the No HTH control and 1 hr Post-fight time points) that subsequently showed increased sway at 2–3 days Post-fight. The dashed lines indicate two balance measures with delayed effects (TMB_Dual_Bal) or acute plus delayed effects (DSB_Bal).

The serum miRNAs we identified with temporal effects tended to show delayed changes, with increases and decreases seen at 2–3 days and 1 week post-fight. Thus, these were examined separately from the saliva miRNAs using PCA on the combined data from 31 total samples. This revealed strong reciprocal loadings for three miRNAs that showed delayed decreases in expression (miR-139-5p, miR-30c-1-3p, miR-421) and six miRNAs (miR-6809-3p, miR-5588-5p, miR-3678-3p, miR-4529-3p, miR3664-3p, and miR-4727-3p) and four functional measures (TSEO, DSB_Bal, TMB_DualBal) that showed delayed increases (**S3 Table; Fig 13**). Notably, one of these functional measures showed an apparent learning effect (TSEO) and one was also identified as highly-associated with acute response salivary miRNAs (DSB_Bal).

#### Validation of NGS data

We performed a direct comparison of the NGS findings with an independent assay of miRNA abundance using the FirePlex technology. We found that: (1) expression levels estimated from purified RNA and direct stabilized saliva were highly-correlated (R = 0.94) in two sample pairs, but that the purified RNA tended to yield higher expression values (**S8A Fig**); (2) there was a robust correlation (median R = 0.78) for the expression levels determined by NGS and FirePlex across 63 pairs of saliva samples that had sufficient material remaining for the validation (**S8B Fig**); (3) the correlation between NGS and FirePlex values was equally robust for 32 sets of samples with sufficient pre- and post-fight data available to examine time-course effects in 8 miRNAs (**S8C Fig**); and (4) the patterns of changes for these 8 miRNAs in the FirePlex data were very consistent with the changes seen in the NGS data on the larger sample set (**S8D Fig**). Notably, the miRNAs that tended to increase relative to pre-fight condition included let-7b-3p, miR-433-3p, miR-30b-5p, and miR-92a-30, while those that decreased at most time points included miR-1307-3p, miR-128-3p, and miR-455-5p.

## Discussion

In the present study, we investigated saliva and serum molecular measures and neurocognitive and balance measures in young adult athletes, both at baseline and various time points following an MMA event, with the goal of establishing diagnostic measures that might accurately predict the likelihood of sports-related concussion based on head impact frequency. This was performed using four complementary approaches. First, we binned subjects on mTBI probability based on the number of hits to the head received during an MMA bout and analyzed a set of potential serum protein biomarkers in a subset of the subjects, based on claims in the existing literature. Our protein data indicated that only one of the potential biomarkers (UCHL1) showed changes that were quantitatively related to the number of hits to the head, while other biomarkers may have shown non-specific increases, potentially due to exercise effects present in subjects who experienced no hits to the head. We then examined serum and salivary miRNA data as well as neurocognitive and balance measures using two-way ANOVA and ROC curve analyses to identify other potential measures which could distinguish low-probability and high-probability TBI samples. Next, we examined the miRNA data using a temporal analysis and revealed molecular biomarkers with either acute or delayed effects relative to the MMA competition. This was true of both saliva and serum miRNAs, although the patterns tended to differ in the two biofluids, with saliva miRNAs responding acutely and serum miRNA exhibiting a delayed response. Because we felt that the most informative biomarkers would be those associated with changes in quantifiable functional measures, we then used PCA analysis of the combined data to delineate temporal patterns in the functional measures related to these miRNAs. This confirmed strong relationships between selected saliva or serum biomarkers and distinct sets of functional measures, which also tended to show acute or delayed effects, despite the presence of practice-related improvement. Overall, our results indicate that studies of molecular and functional biomarkers in mTBI must be rigorously performed and incorporate sensitive measures that are sampled at sufficient frequency to identify potential learning effects in the data. Moreover, our data also indicate that the biomarkers which are most sensitive to mTBI may have strong biological implications. In the discussion that follows, we focus on placing these outcomes in the context of the existing literature.

### Functional outcome measures

Numerous balance measures have been used to evaluate subjects at baseline or following sports related concussion. Our testing included several different types of balance, measures using a computerized accelerometer and tablet device. We also added dual task assessments of balance while subjects were distracted with the requirement to complete a cognitive task, and tasks with purely cognitive demands. Our initial analysis of 14 different measures performed without regard to the timing of the assessments revealed that three measures of balance were potentially sensitive to mTBI likelihood, including the Two Legs Eyes Closed (TLEO) task and two dual tasks including the Digit Span Backwards Balance test (DSB_Bal) and Trail Making B Dual Task Balance test (TMB_Dual_Bal). We also found that the Trail Making A cognitive test (TMA_Cog) was potentially sensitive to mTBI likelihood. While there are many reports in the literature of alterations in balance or neurocognitive function in subjects with mTBI, very few have benefitted from the incorporation of baseline and time-course data. In the present study, the temporal effects on the functional measures were not subjected to a formal repeated measures ANOVA due to the use of mostly different sets of subjects at the different time points and the presence of potential learning effects that would, by their very nature, be subject-dependent. Nonetheless, our PCA analysis of the functional data across time confirmed the presence of significant learning effects in some of the measures, as well as differences in the time point which demonstrated the largest change. These observations suggest that some balance measures, particularly those involving high dual-task cognitive demands, such as the TMB_Dual_Bal and DSB_Bal, may reveal their maximal effects at a somewhat delayed time point rather than acutely (**Fig13**). In contrast, the acute time point assessments that were performed within an hour of the MMA fight indicated that the most sensitive and reliable measures included several simple balance measures (e.g., TSECFP) as well as cognitive measures (TMA_Cog, TMB_Dual_Cog) (**Fig 12**). While other balance tests did reveal an increase in body sway post-fight relative to immediately pre-fight, they also demonstrated varying degrees of overall decreased sway across time, particularly the TLEOFP, which we believe represents a learning effect. Improvement in this task performance might not be surprising given the ability of subjects to use visual feedback signals to help adjust their postural stability. In contrast, the TSECFP task likely represents the most difficult task and subjects can only use proprioceptive cues but not visual information, and this did not demonstrate any apparent improvement across time.

The trail making A and B tests have been widely used to assess cognitive performance, and recent studies have implemented computerized versions of these tests for examining performance in subjects with mTBI. For example, Woods and colleagues [15] examined the evidence for practice-related learning and sensitivity to TBI using a computerized version of the trail making A and B tests in separate cohorts of subjects. Notably, they found evidence of a significant learning effect in the trail making B test, but not the A test, although they also claimed that both tests were sensitive to TBI. Our data strongly support their findings, but also indicate that there may be an optimal time point for examination of trail making performance in subjects who have had prior exposure to the test.

### Molecular outcome measures

#### Protein biomarkers

Numerous studies in both human subjects and rodent models have examined the potential utility of different serum proteins in the context of mTBI and more commonly severe TBI. The results of these studies have been extensively reviewed [16, 17]. Based on these findings, we examined a set of 11 potential biomarkers in a subset of our MMA fighter samples, obtained immediately pre- and post-fight. While some of these proteins showed elevations post-fight relative to pre-fight, this was largely true regardless of whether subjects experienced many (or any) hits to the head. The only exception to this was UCHL1, which showed an increase post-fight that was correlated with the number of hits to the head. Interestingly, although the literature on UCHL1 contains many reports of changes in different studies, this is not a uniform finding and many studies have also claimed decreases in expression or a lack of change following mTBI [16, 17]. Our data indicate that the increased expression of UCHL1 in the serum may only be observed in the most severe cases of mTBI (i.e., MMA fighters with 30 or more hits to the head). Notably, a blood test for concussion was recently approved by the Food and Drug Administration involving measures of UCHL1 and GFAP (https://www.fda.gov/newsevents/newsroom/pressannouncements/ucm596531.htm).

#### miRNA biomarkers

There have been several human studies published on potential blood or other biofluid measures of mTBI using miRNAs, including recent work on TBI in teenage children [12, 14]. These studies have generally focused on examination of a single time point in a cross-sectional comparison of mTBI and control subjects, or on focused examination of a small number of miRNAs across multiple time points. Very few studies have utilized exercise- or non-head injury (e.g., musculoskeletal injury controls in mTBI). Other studies in laboratory animals have generally involved rodents, and often employed multiple timepoints or open TBI procedures more analogous to severe TBI. Open procedures clearly introduce conditions that are beyond the scope of what occurs in mild TBI. Our study attempted to explore the issues of mTBI severity and time on the miRNA data and place the changes within the context of the functional data and previous findings in the field.

The majority of our candidate miRNA biomarkers have not been reported in the previous literature. It is likely that our use of a baseline timepoint to normalize each miRNA and functional outcome measure produced greater sensitivity for detection. However, several of our candidate mTBI biomarkers have been previously reported. These miRNA biomarkers can be specified as exact matches or highly-related matches that derive from the same miRNA gene. Among the miRNAs that we detected with changes related to the hits to the head, 12 were novel and 9 are exact matches or highly-related to those identified in previous studies of TBI. Among the miRNAs with definitive time-course changes in our data, 17 were novel and 7 were exact matches or are highly-related to those reported in previous studies of TBI (**S4 Table**). Notably, three of the current miRNAs we identified were the same and three were highly-related to those previously reported as changed in saliva from children with mild TBI [12, 13](**S4 Table**). Notably, it is likely that differences in the findings from those reports compared with the present study may have arisen due to the much later collection times (6 days post-injury) as well as differences in subject age and the mechanism of injury. Nonetheless, some of the same and related miRNAs were still identified. Moreover, several of the exact and highly-related matches were also found in studies of TBI that sampled peripheral blood in humans or rodents, as well as human CSF or rodent brain tissue (**S4 Table**).

#### Biomarker routes of transfer

We are highly interested in the trafficking of miRNAs between the central nervous system (CNS) and peripheral locations. Because blood brain barrier (BBB) disruption occurs in all levels of TBI severity, it is generally understood that serum biomarkers can serve as an indirect readout of pathological processes occurring in the CNS of affected individuals. What is less apparent, however, is how changes in brain function could be reflected in saliva. Two potential routes are worth noting. First, the brain stem provides a potential CNS-to-oral cavity route via the sensory (V, VII, IX) and motor (XII, X, XII) nerves that innervate the salivary glands and tongue. A similar mechanism of transmission from CNS to saliva occurs in Rabies virus infection, wherein the virus travels from muscle, to brain, and eventually to the cranial nerves that innervate the salivary glands. A second route for likely miRNA delivery to the mouth involves slow transport via the glymphatic system, which has been strongly implicated in CNS responses to TBI [18, 19].

#### Biological pathways of miRNA biomarkers of HTH

Our unbiased pathway-based analysis of the miRNAs most strongly-related to hits to the head, implicated several with potential relevance to TBI. Four notable ones included Ubiquitin-mediated proteolysis (which would be involved in degradation of proteins), Transforming growth factor-beta (TGF-beta) (involved in anti-inflammatory responses), and Axon guidance and Glutamatergic synapse (involved in promoting normal brain communication and connectivity (**Table 7**). Within each of these pathways, the most common patterns seen were decreases in salivary miRNAs and a mix of increases or decreases in serum miRNAs. Because of this bidirectionality, it is not possible to infer direct cause and effect relationships between our findings and the overall impact on a particular pathway. Nonetheless, careful analysis of individual miRNAs can be revealing.

For example, review of the literature indicated that several of our miRNAs were implicated in TBI-related processes including BBB disruption, neuroinflammation, and neurodegenerative disease. Notably, the miR-155 family, in particular, appears to influence all three of these processes. Evidence suggests that miR-155 is a key modulator of inflammation and contributes to proinflammatory signaling via targeted repression of anti-inflammatory molecules including suppressor of cytokine signaling-1 (SOCS-1) [20] and Src homology-2 domain-containing inositol 5 phosphatase 1 (SHIP1) [21]. miR-155-5p is increased in the serum of MMA fighters in our study, as well as in the hippocampus of rat and mouse models of moderate TBI (**S4 Table**). miR-155 is likewise elevated in neuroinflammatory disorders including Alzheimer’s disease [22], amyotrophic lateral sclerosis [23], multiple sclerosis (MS) [24], and Parkinson’s disease [25]. miR-155 is also upregulated at the neurovascular unit of active MS lesions, and is reported to negatively regulate BBB function during neuroinflammation associated with experimental autoimmune encephalomyelitis, an animal model of MS [26]. In that study, miR-155 modulated brain endothelial barrier function by targeting both cell-cell (annexin-2 and claudin-1) and cell-matrix (DOCK-1 and syntenin-1) interactions. miR-155-5p deletion was also shown to reduce inflammatory signaling in macrophages, and to enhance their ability to support neuron survival and axon growth in macrophage/neuron co-cultures [27]. Thus, the alteration in miR-155-5p we detected may represent a hallmark of delayed proinflammatory responses to BBB disruption.

Another individual miRNA, miR-20a-5p, was also altered (elevated) in our study where it showed a relationship to HTH. This miRNA has also been reported as elevated in the serum of human subjects with TBI and in various CNS and PNS injury models [13] [28–41] (**S4 Table**). Its close relative, miR-20a, is upregulated in a murine model of traumatic spinal cord injury. Interestingly, infusion of miR-20a into the uninjured spinal cord induced inflammation and motor neuron degeneration, and inhibiting miR-20a *in vivo* increased neuronal survival and promoted neurogenesis that corresponded with rescued expression of a defined target gene, neurogenin 1 (Ngn1), a transcription factor involved in neuronal differentiation [42]. Thus, miR-20a may also represent a common response to CNS or PNS injury, that could lead to undesired outcomes (inflammation and neurodegeneration) following TBI.

#### Biological pathways of temporally-mediated miRNA biomarkers of TBI

Our pathway-based analysis of the miRNAs that showed the greatest temporal responses to a head impact also implicated several pathways of relevance for TBI. For example, the acute salivary response (ASR) miRNAs and delayed serum response (DSR) miRNAs were both enriched for gene targets involved in Glutamatergic synapse, Axon guidance, ErbB signaling, Thyroid hormone synthesis or signaling, as well as Long-term depression or potentiation (**Tables 10 and 11**). In the case of the pathways targeted by ASRs, it can be inferred that this response would impair the normal function of these pathways (since the biomarkers were all increased in saliva). However, the responses in the serum were bi-directional, so a general conclusion is not supported by inspection of the DSRs, and detailed inspection of individual miRNAs and the functional importance of their specific targets will be required, which is outside the scope of this report. Nonetheless, we can point to at least one of the ASR biomarkers that might be involved in a protective or adaptive response. For example, let-7b-3p, which was acutely elevated in the saliva in our study, was also shown to be upregulated in human brain endothelial cells by treatment with proinflammatory cytokines TNFα and IFNγ. Because this treatment significantly impairs the cell monolayer barrier function [26], it suggests that let-7b-3p, unlike miR-155, may play a role in maintaining a tight BBB following TBI. Additional support for the relative importance of let-7b-3p is available from the study of salivary biomarkers of TBI in pediatric populations [13], who reported that let-7b-5p was reduced 4-fold in mild TBI and associated with fatigue symptoms 4 weeks post-concussion. These seemingly disparate observations can be reconciled when considering the role of arm selection during miRNA maturation. Individual cells largely favor either -3p or -5p selection, but not both. Thus, the decrease in let-7b-5p reported in the previous study can be viewed as complementary to the increase in let-7b-3p we observed. Another possibility is that there is a complex temporal pattern of responses which we did not discern.

#### Non-specific effects

The present study attempted to control for the potential influence of individual variation as well as exercise-related exertion and non-head impact injuries on the biomarker outcomes, by first normalizing all the miRNA data into changes in expression compared to a pre-fight baseline and examining these changes using control samples who had either 0–3 hits to the head (for the HTH analysis) or no hits to the head (for the temporal analysis). Notably, the control subjects also included some samples from subjects whose fight was cancelled on-site. By including these samples, we believe that we have at least partly mitigated the influence of non-specific exercise-related effects, as well as non-head impact effects that might occur to other parts of the body (e.g., musculoskeletal injuries). Nonetheless, future studies using more fighters and potentially athletes engaged in other contact sports would be helpful for determining the generalizability of our present results.

#### Relationship to functional measures

Examination of the miRNAs that were identified in our HTH-based analysis indicated only very modest associations with any of the functional outcome measures (as opposed to the HTH levels themselves, which was the basis for the subject and sample binning) (**Table 8**). The one possible exception to this was miR-4766-5p, which showed a significant negative correlation with the body sway measures in the subset of subjects who provided a serum sample post-fight. However, this is difficult to interpret given that that there was no meaningful association between salivary levels of miR-4766-5p with body sway measures in the subjects who provided a saliva sample. A select few other miRNAs did show fairly robust associations between their expression changes in both biofluids and the HTH values. The best example is miR-6770-5p, which showed a positive correlation that survived FDR correction (**Table 8**). Unfortunately, there are no reports in the literature pertaining to TBI for this miRNA. The top predicted targets of this gene (according to TargetScan) are also inconclusive.

In contrast, several of the miRNAs that showed temporal patterns in their changes were at least partly related to the functional measures that were obtained. For example, among salivary ASRs, let-7b-3p had strong negative loading on Factor 1 (-0.622), while miR-3118, miR-3170, miR-3919, miR-433-3p, miR-4632-3p, and miR-6870-3p all had strong positive loadings on Factor 1 (>0.683) (**S2 Table**). This occurred along with strong negative loadings (< -0.4) for two functional measures (TMA_Cog and TMB_Dual_COG), and strong positive loadings (> 0.4) for three body sway measures (TLEOFP, TSEOFP, DSB_Bal). Thus, changes in let-7b-3p are positively correlated with cognitive function, while changes in at least 6 salivary ASRs appear to be positively associated with impairment of balance. Inspection of the serum DSRs leads to similar inferences about a different set of biomarkers. miRNAs miR-139-5p and miR-421 showed strong negative weights (< -0.4) on Factor 1 which was also seen for four measures of balance, including TSEO, TLEOFP, TMB_Dual-Bal, and DSB_Bal (**S3 Table**). Thus, these miRNAs are positively associated with impairment of balance, whereas miRNAs miR-3664-3p, miR-3678-3p, miR-4629-3p, miR-4727-3p, miR-5588-5p, and miR-6809-3p all showed strong positive weights (> 0.4) on Factor 1 and are thus negatively or inversely associated with impaired balance. Interestingly, no serum DSR miRNAs were strongly related to cognitive outcome measures.

## Limitations

It is important to point out a number of limitations in the present study. First, subjects were not systematically evaluated for general symptoms of concussion and mental responsiveness following the fight using a standardized assessment tool (such as the SCAT). Second, we were not able to use a full factorial model in our analysis to formally investigate the effect of biofluid and time of sampling due to the use of slightly different sets of subjects across time. Third, although we quantified the hits to the head experienced during the MMA fight, there is considerable heterogeneity in the force of each head impact, depending on the fighters themselves and the trajectory and location of the blow to the head. Finally, the data which were obtained may not be fully representative of what might occur in the general or young adult population because they were obtained from amateur mixed martial arts fighters. Notably, however, we do point out that every subject in the study was employed in a full-time job or attended school, and only participated in MMA fighting as an athletic hobby. Thus, this particular group of amateur MMA fighters may be fairly representative of young adult athletes who engage in contact sports.

## Conclusions

Our analysis has revealed a number of important findings for the mTBI biomarker field. First, several salivary and serum miRNAs are robustly altered after a sport-related head impact. Some of these miRNAs show association with the quantity of head impacts, and affect processes involved in both adaptive and maladaptive responses. Other miRNAs show changes that differed across time, which underscores the importance of designing studies to capture the time course of biomarker responses. Both the HTH-related and temporally-related sets of miRNAs are predicted to alter biological processes that are potentially highly-relevant for TBI studies. Furthermore, the changes in some of these same miRNAs also show associations with computerized functional outcome measures of both balance and cognitive function. In contrast, our limited analysis of potential protein biomarkers did not yield strong associations with the number of head impacts, with the possible exception of UCHL1. Clearly, much additional work needs to be done to determine the best molecular biomarkers of mTBI from peripheral biofluids and to relate these to the most sensitive functional measures.

## Supporting information

S1 Fig Serum protein changes compared with hits to the head (HTH). For each of the 9 proteins, the change post-fight compared to pre-fight is expressed as a percentage of the pre-fight level and plotted on the Y-axis. The X-axis indicates the HTH values counted by an independent viewer of a video recording of each MMA fight. Note that none of these proteins displayed strong associations with HTH, with maximal r^2^ values less than 0.09.
(PDF)

S2 Fig Enrichment of changed miRNAs for target genes in the KEGG Ubiquitin-mediated proteolysis pathway. In this pathway, 80 genes were targeted by a total of 19 miRNAs. Genes targeted by 1 miRNA are shown in yellow, and genes targeted more than 1 miRNA are shown in orange. Genes in green have miRNAs that are predicted to target them but none of these were contained in the list of 21 changed miRNAs. Genes in white do not have predicted miRNAs that target them. Adapted with permission from KEGG: Kyoto Encyclopedia of Genes and Genomes [43].
(PDF)

S3 Fig Enrichment of changed miRNAs for target genes in the KEGG TGF-beta signaling pathway. Conventions same as S2 Fig. This pathway contained 46 genes that were predicted to be targeted by 20 miRNAs. Adapted with permission from KEGG: Kyoto Encyclopedia of Genes and Genomes [43].
(PDF)

S4 Fig Enrichment of changed miRNAs for target genes in the KEGG Axon guidance pathway. Conventions same as S2 Fig. This pathway contained 70 genes that were predicted to be targeted by 17 miRNAs. Adapted with permission from KEGG: Kyoto Encyclopedia of Genes and Genomes [43].
(PDF)

S5 Fig Enrichment of changed miRNAs for target genes in the KEGG Glutamatergic synapse pathway. Conventions same as S2 Fig. This pathway contained 61 genes that were predicted to be targeted by 20 miRNAs. Adapted with permission from KEGG: Kyoto Encyclopedia of Genes and Genomes [43].
(PDF)

S1 Table Temporal miRNAs, indicating biofluid & directional change. Of the 47 miRNAs with significant effects of Time, 12 were acutely increased in saliva, 5 had delayed decreases in serum and 8 had delayed increases in serum post-fight.
(DOCX)

S6 Fig Enrichment of changed miRNAs for target genes in the KEGG Glutamatergic synapse pathway. Conventions same as S2 Fig. Note that both saliva miRNAs and serum miRNAs target many of the same genes in this pathway. Adapted with permission from KEGG: Kyoto Encyclopedia of Genes and Genomes [43].
(PDF)

S7 Fig Enrichment of temporally-regulated miRNAs in pathways involved in learning and memory from the saliva. Pathways shown are Long-term depression for saliva (upper) and Long-term potentiation for serum (lower). Same conventions as S2 Fig. Adapted with permission from KEGG: Kyoto Encyclopedia of Genes and Genomes [43].
(PDF)

S8 Fig Validation of changes in salivary miRNA using FirePlex assays. **A**, Examination of performance of FirePlex assay [44] in stabilized saliva compared with purified RNA. Note the high correlation between the two, but somewhat greater expression for miRNAs expression at moderate levels in the purified RNA. **B**, Bean plot of Spearman correlation rho values directly comparing NGS normalized and FirePlex normalized data for 64 pairs of samples interrogating 15 miRNAs at a range of 4 sample times. **C**, Comparison of median expression of 15 miRNAs in FirePlex and NGS data for 32 pairs of samples with pre- and post-fight data available. **D**, Changes in expression (relative to the pre-fight timepoint) in the 32 samples from panel C for 8 miRNAs predicted to change in specific directions following the fight based on the NGS data. Five miRNAs predicted to be increased from the NGS data are shown in red. Three miRNAs predicted to decrease are in green. Note that the specific predictions do not apply to all of the post-fight timepoints. Most of these miRNAs showed patterns of changes that were consistent with the NGS changes for several of the time points.
(PDF)

S2 Table Factor weights from PCA of ASR miRNAs and functional data. Values shown indicate factor loading scores.
(DOCX)

S3 Table Factor weights from PCA of DSR miRNAs and functional data. (DOCX)

S4 Table miRNAs with significant effect of HTH (Table 5) or defined temporal effects (S1 Table) that have been previously reported in TBI studies. Upper indicates exact miRNA matches in previous studies. Lower indicates highly-related miRNA matches in previous studies. HTH, changes related to hits to the head in current study; T, time-course changes in current study. We note that miR-155-5p was decreased in severe TBI as determined by microarray analysis in one of the studies, but failed to show differential expression in qRT-PCR validation assay. Similarly, miR-455-3p was decreased in mild TBI as determined by microarray analysis, but failed to show differential expression in qRT-PCR validation assay.
(DOCX)
