# Supplementary material for: Comparison of serum and saliva miRNAs for identification and characterization of mTBI in adult mixed martial arts fighters
Source: PLoS One. 2019 Jan 2;14(1):e0207785. doi: 10.1371/journal.pone.0207785 (PMC6314626; doi:10.1371/journal.pone.0207785)
Supplement: S1 Fig — For each of the 9 proteins, the change post-fight compared to pre-fight is expressed as a percentage of the pre-fight level and plotted on the Y-axis. The X-axis indicates the HTH values counted by an independent viewer of a video recording of each MMA fight. Note that none of these proteins displayed strong associations with HTH, with maximal r2 values less than 0.09. (PDF) [file pone.0207785.s001.pdf]

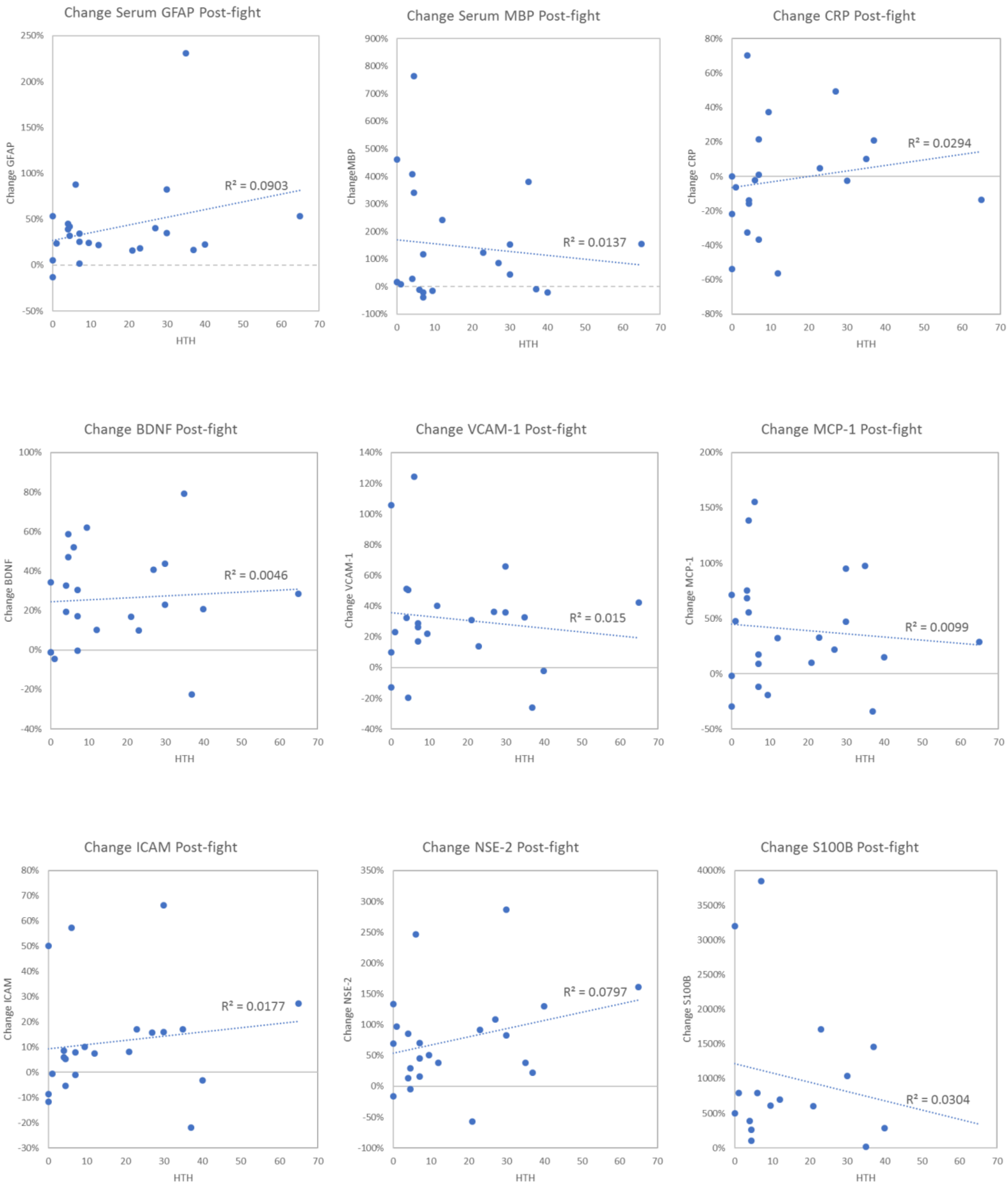
Change Serum GFAP Post-fight
Change GFAP
R² = 0.0903
HTH
Change Serum MBP Post-fight
ChangeMBP
R² = 0.0137
HTH
Change CRP Post-fight
Change CRP
R² = 0.0294
HTH
Change BDNF Post-fight
Change BDNF
R² = 0.0046
HTH
Change VCAM-1 Post-fight
Change VCAM-1
R² = 0.015
HTH
Change MCP-1 Post-fight
Change MCP-1
R² = 0.0099
HTH
Change ICAM Post-fight
Change ICAM
R² = 0.0177
HTH
Change NSE-2 Post-fight
Change NSE-2
R² = 0.0797
HTH
Change S100B Post-fight
Change S100B
R² = 0.0304
HTH
