# Supplementary material for: Comparison of serum and saliva miRNAs for identification and characterization of mTBI in adult mixed martial arts fighters
Source: PLoS One. 2019 Jan 2;14(1):e0207785. doi: 10.1371/journal.pone.0207785 (PMC6314626; doi:10.1371/journal.pone.0207785)
Supplement: S2 Fig — In this pathway, 80 genes were targeted by a total of 19 miRNAs. Genes targeted by 1 miRNA are shown in yellow, and genes targeted more than 1 miRNA are shown in orange. Genes in green have miRNAs that are predicted to target them but none of these were contained in the list of 21 changed miRNAs. Genes in white do not have predicted miRNAs that target them. Adapted with permission from KEGG: Kyoto Encyclopedia of Genes and Genomes [43]. (PDF) [file pone.0207785.s002.pdf]

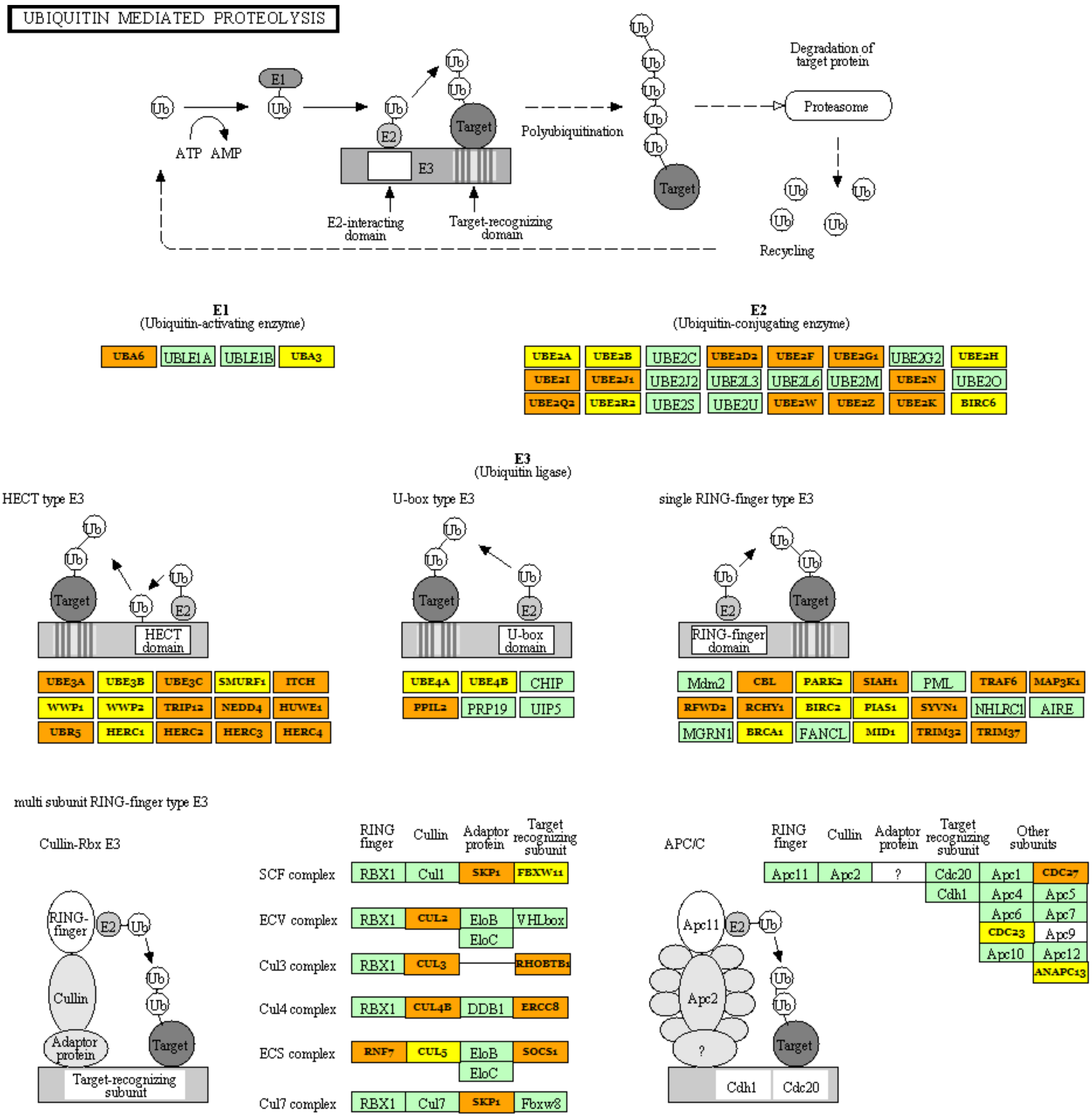
UBIQUITIN MEDIATED PROTEOLYSIS
Ub
E1
ATP AMP
E2
Target
E3
E2-interacting domain
Target-recognizing domain
Polyubiquitination
Degradation of target protein
Proteasome
Recycling
E1
(Ubiquitin-activating enzyme)
UBA6
UBLE1A
UBLE1B
UBA3
E2
(Ubiquitin-conjugating enzyme)
UBE2A
UBE2B
UBE2C
UBE2D2
UBE2F
UBE2G1
UBE2G2
UBE2H
UBE2I
UBE2J1
UBE2J2
UBE2L3
UBE2L6
UBE2M
UBE2N
UBE2O
UBE2Q2
UBE2R2
UBE2S
UBE2U
UBE2W
UBE2Z
UBE2K
BIRC6
E3
(Ubiquitin ligase)
HECT type E3
HECT domain
UBE3A
UBE3B
UBE3C
SMURF1
ITCH
WWP1
WWP2
TRIP12
NEDD4
HUWE1
UBR5
HERC1
HERC2
HERC3
HERC4
U-box type E3
U-box domain
UBE4A
UBE4B
CHIP
PPIL2
PRP19
UIP5
single RING-finger type E3
RING-finger domain
Mdm2
CBL
PARK2
SIAH1
PML
TRAF6
MAP3K1
RFWD2
RCHY1
BIRC2
PIAS1
SYVN1
NHLRC1
AIRE
MGRN1
BRCA1
FANCL
MID1
TRIM32
TRIM37
multi subunit RING-finger type E3
Cullin-Rbx E3
RING-finger
Cullin
Adaptor protein
Target-recognizing subunit
RING finger
Cullin
Adaptor protein
Target recognizing subunit
SCF complex
RBX1
Cul1
SKP1
FBXW11
ECV complex
RBX1
CUL2
EloB
VHLbox
EloC
Cul3 complex
RBX1
CUL3
RHOBTB1
Cul4 complex
RBX1
CUL4B
DDB1
ERCC8
ECS complex
RNF7
CUL5
EloB
SOCS1
EloC
Cul7 complex
RBX1
Cul7
SKP1
Fbxw8
APC/C
RING finger
Cullin
Adaptor protein
Target recognizing subunit
Other subunits
Apc11
Apc2
?
Cdc20
Apc1
CDC27
Cdh1
Apc4
Apc5
Apc6
Apc7
CDC23
Apc9
Apc10
Apc12
ANAPC13
Cdh1
Cdc20
