# Supplementary figures and images for: Comparison of serum and saliva miRNAs for identification and characterization of mTBI in adult mixed martial arts fighters
Source: PLoS One. 2019 Jan 2;14(1):e0207785. doi: 10.1371/journal.pone.0207785 (PMC6314626; doi:10.1371/journal.pone.0207785)

# TGF-BETA SIGNALING PATHWAY

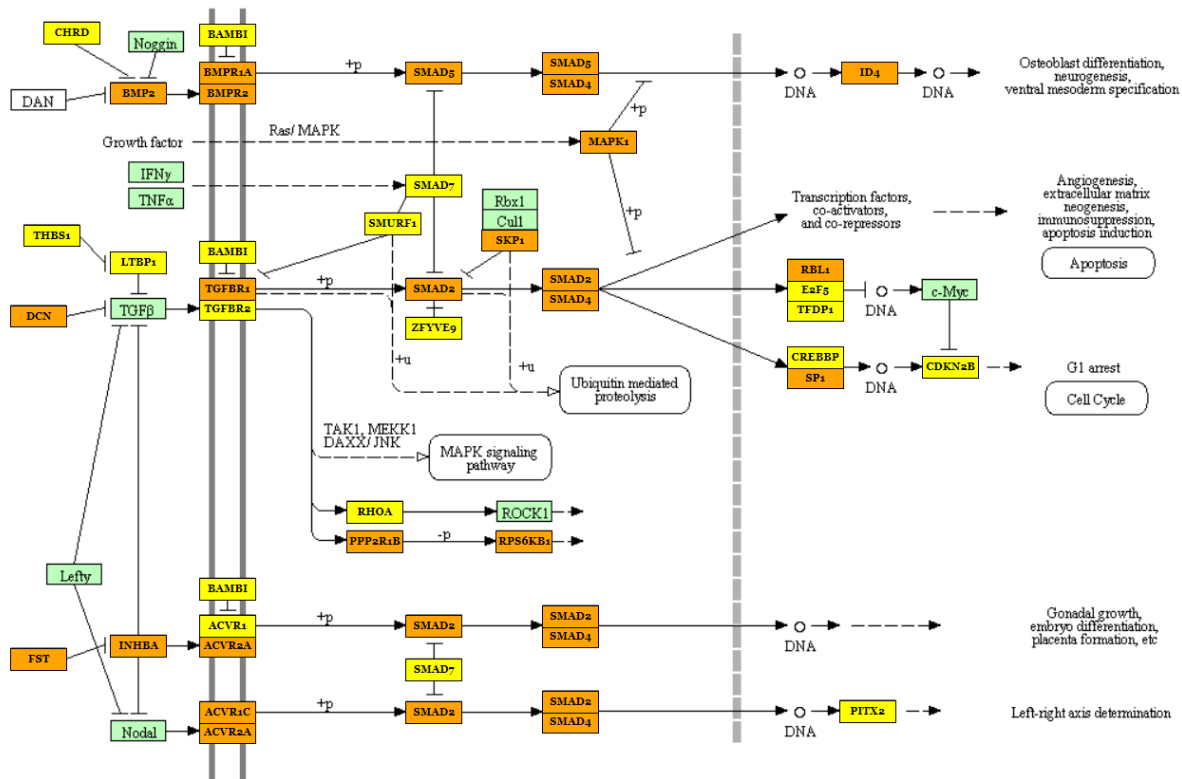

Supplement: S3 Fig — Conventions same as S2 Fig. This pathway contained 46 genes that were predicted to be targeted by 20 miRNAs. Adapted with permission from KEGG: Kyoto Encyclopedia of Genes and Genomes [43]. (PDF) [file pone.0207785.s003.pdf]

# AXON GUIDANCE

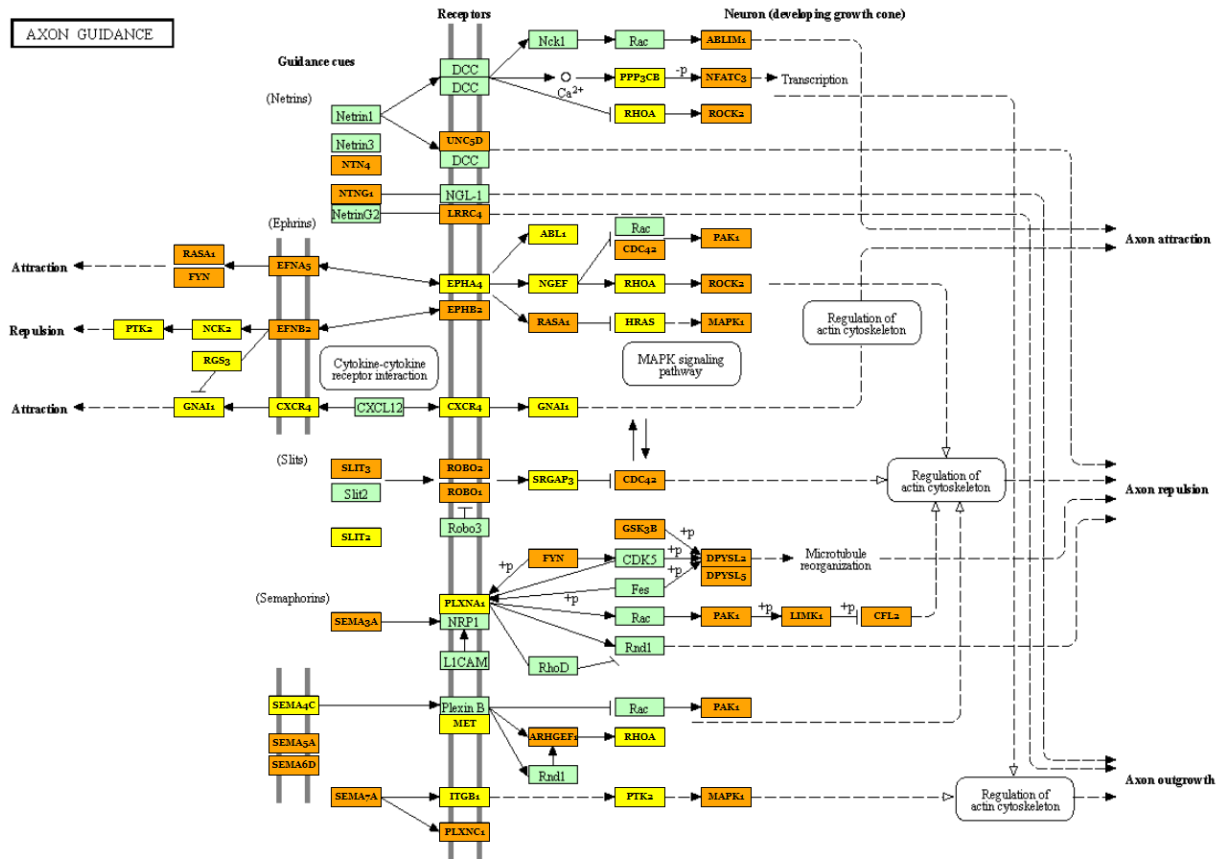

Supplement: S4 Fig — Conventions same as S2 Fig. This pathway contained 70 genes that were predicted to be targeted by 17 miRNAs. Adapted with permission from KEGG: Kyoto Encyclopedia of Genes and Genomes [43]. (PDF) [file pone.0207785.s004.pdf]

# GLUTAMATERGIC SYNAPSE

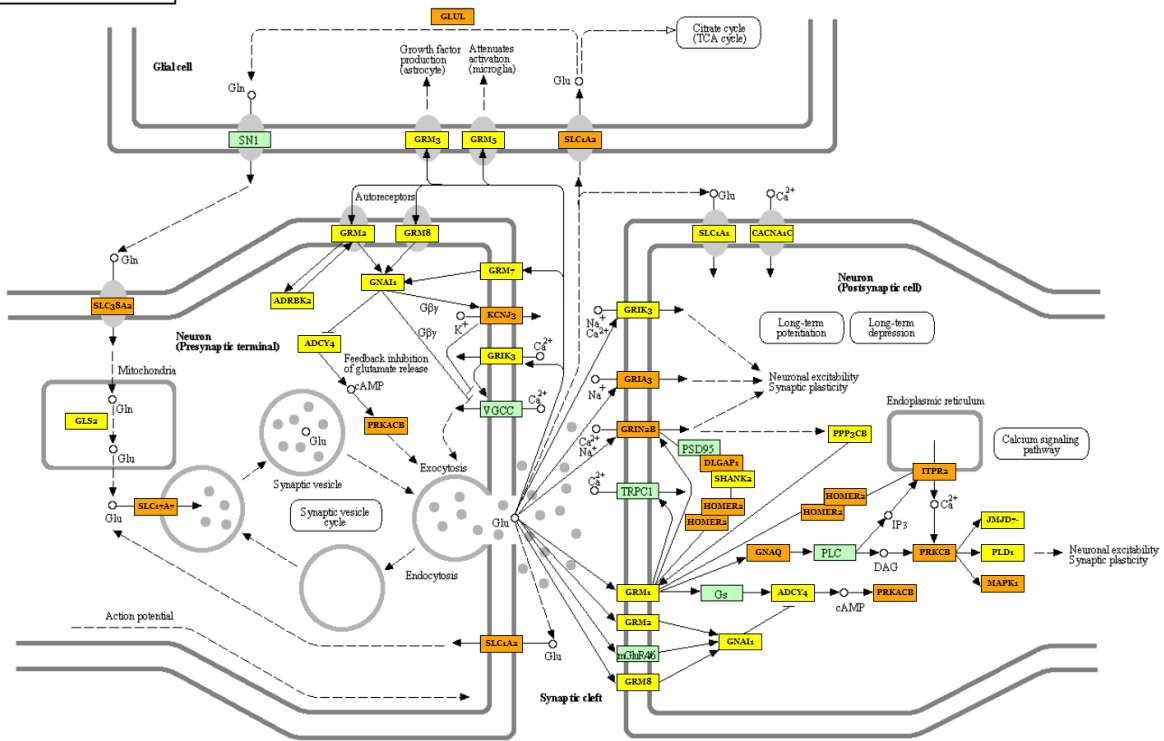

Supplement: S5 Fig — Conventions same as S2 Fig. This pathway contained 61 genes that were predicted to be targeted by 20 miRNAs. Adapted with permission from KEGG: Kyoto Encyclopedia of Genes and Genomes [43]. (PDF) [file pone.0207785.s005.pdf]

## GLUTAMATERGIC SYNAPSE

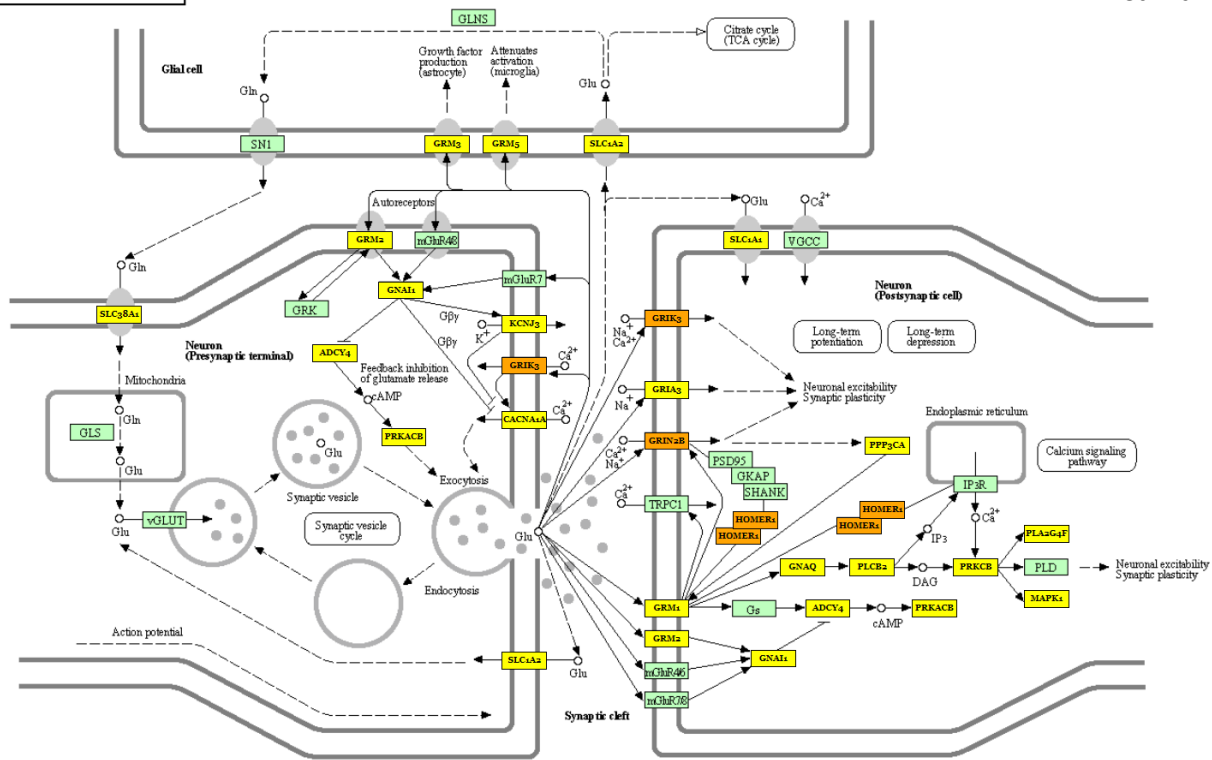

## GLUTAMATERGIC SYNAPSE

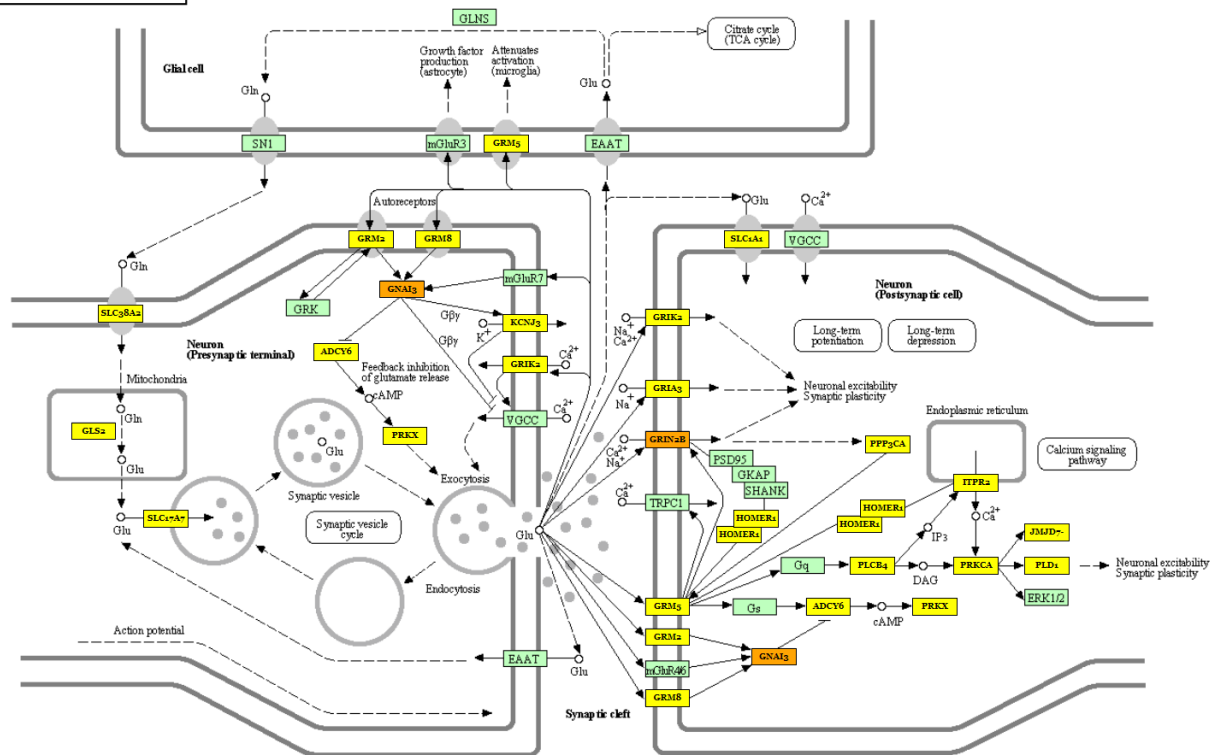

Supplement: S6 Fig — Conventions same as S2 Fig. Note that both saliva miRNAs and serum miRNAs target many of the same genes in this pathway. Adapted with permission from KEGG: Kyoto Encyclopedia of Genes and Genomes [43]. (PDF) [file pone.0207785.s007.pdf]

# LONG-TERM DEPRESSION

Saliva

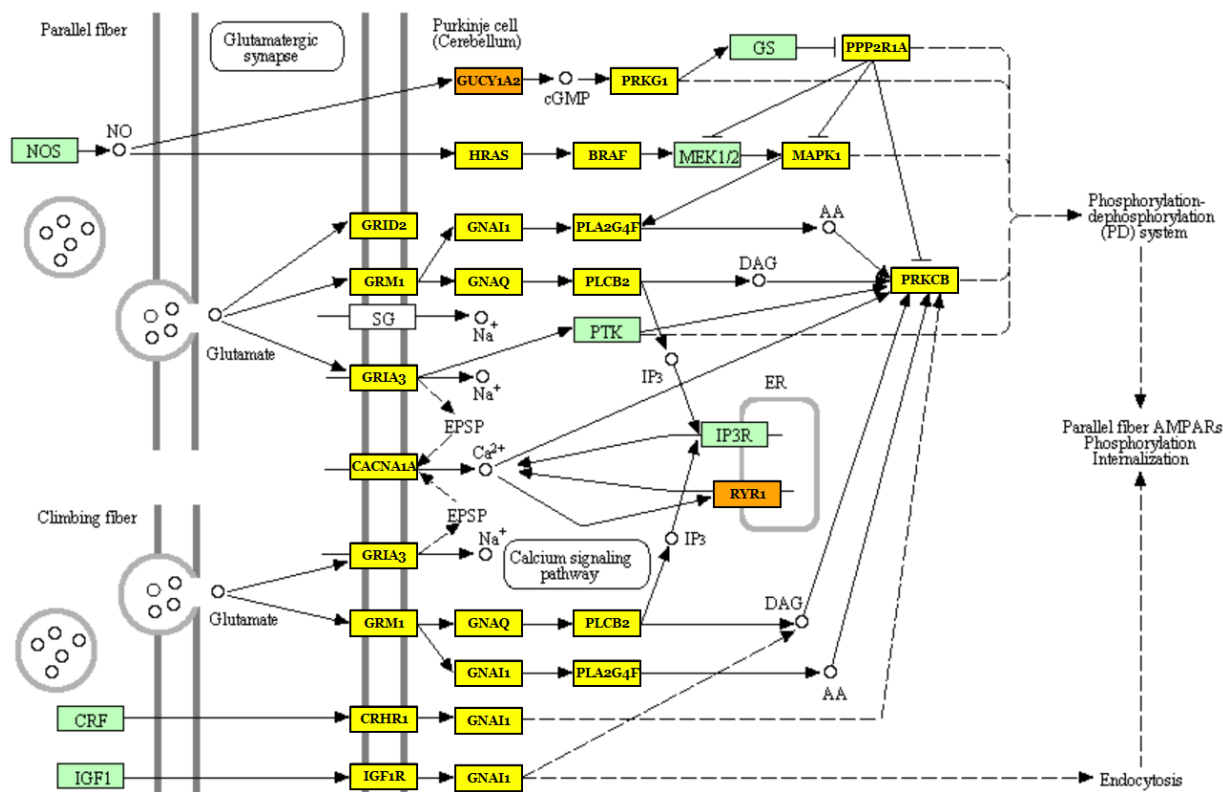

# LONG-TERM POTENTIATION

Serum

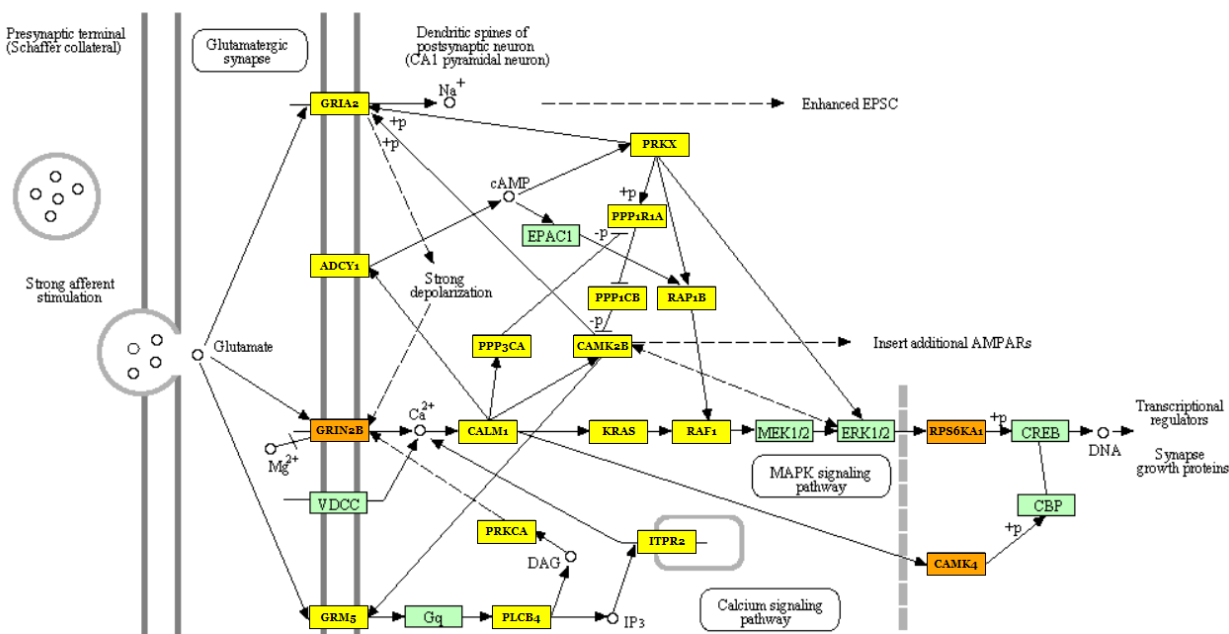

Supplement: S7 Fig — Pathways shown are Long-term depression for saliva (upper) and Long-term potentiation for serum (lower). Same conventions as S2 Fig. Adapted with permission from KEGG: Kyoto Encyclopedia of Genes and Genomes [43]. (PDF) [file pone.0207785.s008.pdf]

**A**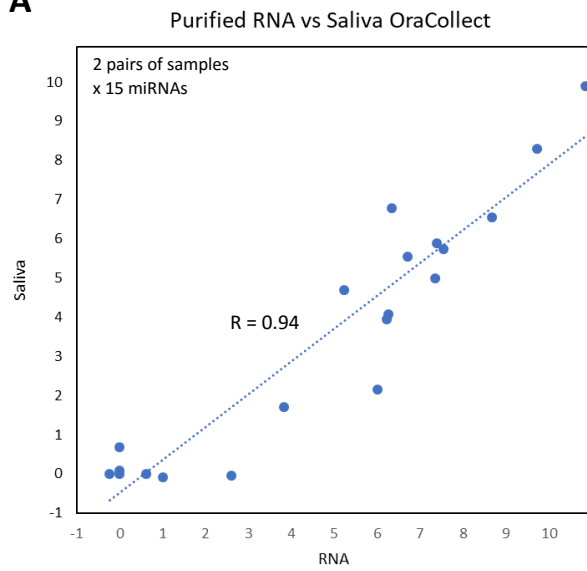**B**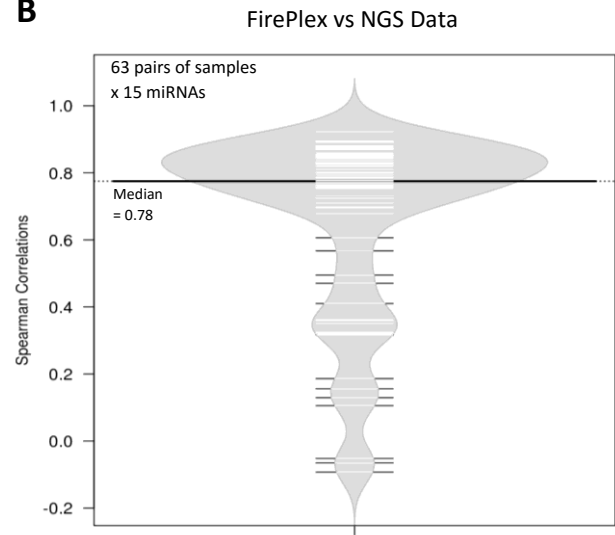**C**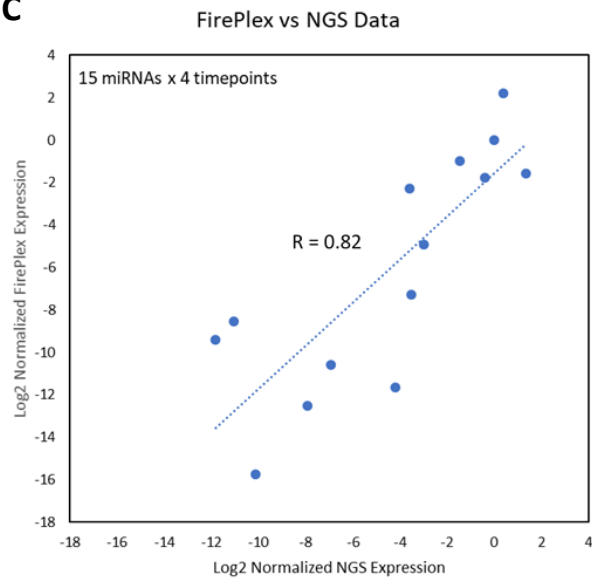**D**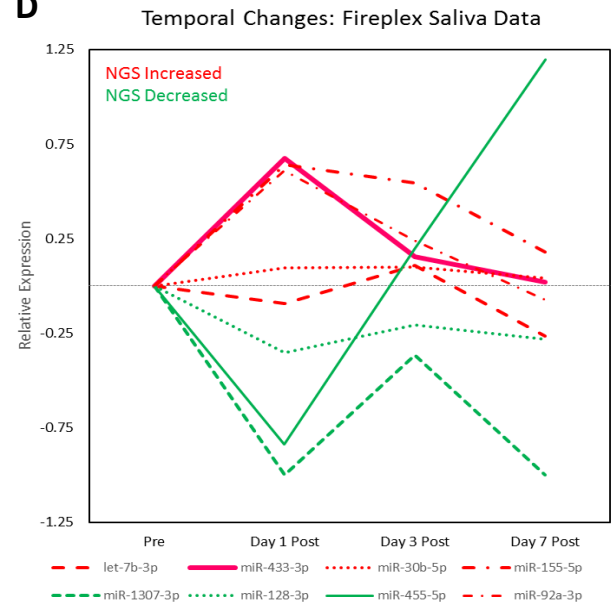

Supplement: S8 Fig — A, Examination of performance of FirePlex assay [44] in stabilized saliva compared with purified RNA. Note the high correlation between the two, but somewhat greater expression for miRNAs expression at moderate levels in the purified RNA. B, Bean plot of Spearman correlation rho values directly comparing NGS normalized and FirePlex normalized data for 64 pairs of samples interrogating 15 miRNAs at a range of 4 sample times. C, Comparison of median expression of 15 miRNAs in FirePlex and NGS data for 32 pairs of samples with pre- and post-fight data available. D, Changes in expression (relative to the pre-fight timepoint) in the 32 samples from panel C for 8 miRNAs predicted to change in specific directions following the fight based on the NGS data. Five miRNAs predicted to be increased from the NGS data are shown in red. Three miRNAs predicted to decrease are in green. Note that the specific predictions do not apply to all of the post-fight timepoints. Most of these miRNAs showed patterns of changes that were consistent with the NGS changes for several of the time points. (PDF) [file pone.0207785.s009.pdf]
