# Supplementary material for: Comparison of serum and saliva miRNAs for identification and characterization of mTBI in adult mixed martial arts fighters
Source: PLoS One. 2019 Jan 2;14(1):e0207785. doi: 10.1371/journal.pone.0207785 (PMC6314626; doi:10.1371/journal.pone.0207785)
Supplement: S1 Table — Of the 47 miRNAs with significant effects of Time, 12 were acutely increased in saliva, 5 had delayed decreases in serum and 8 had delayed increases in serum post-fight. (DOCX) [file pone.0207785.s006.docx]

**Supporting Table S1. Temporal miRNAs, indicating biofluid & directional change**

| **miRNA** | **Acute Saliva ↑** | **Delayed Serum ↑** | **Delayed Serum ↓** |
| --- | --- | --- | --- |
| hsa-let-7b-3p | x |  |  |
| hsa-miR-30c-1-3p |  | x |  |
| hsa-miR-139-5p |  | x |  |
| hsa-miR-421 |  | x |  |
| hsa-miR-433-3p | x |  |  |
| hsa-miR-501-3p |  | x |  |
| hsa-miR-550a-3-5p |  | x |  |
| hsa-miR-601 | x |  |  |
| hsa-miR-608 | x |  |  |
| hsa-miR-1270 |  |  | x |
| hsa-miR-2682-5p | x |  |  |
| hsa-miR-3118 | x |  |  |
| hsa-miR-3170 | x |  |  |
| hsa-miR-3664-3p |  |  | x |
| hsa-miR-3678-3p |  |  | x |
| hsa-miR-3919 | x |  |  |
| hsa-miR-4529-3p |  |  | x |
| hsa-miR-4632-3p | x |  |  |
| hsa-miR-4660 | x |  |  |
| hsa-miR-4727-3p |  |  | x |
| hsa-miR-4760-5p | x |  |  |
| hsa-miR-5588-5p |  |  | x |
| hsa-miR-6809-3p |  |  | x |
| hsa-miR-6870-3p | x |  |  |
| hsa-miR-8089 |  |  | x |

Of the 47 miRNAs with significant effects of Time, 12 were acutely increased in saliva,

5 had delayed decreases in serum and 8 had delayed increases in serum post-fight.
