# Supplementary material for: Comparison of serum and saliva miRNAs for identification and characterization of mTBI in adult mixed martial arts fighters
Source: PLoS One. 2019 Jan 2;14(1):e0207785. doi: 10.1371/journal.pone.0207785 (PMC6314626; doi:10.1371/journal.pone.0207785)
Supplement: S2 Table — Values shown indicate factor loading scores. (DOCX) [file pone.0207785.s010.docx]

| **Measure** | **Factor 1** | **Factor 2** | **Factor 3** |
| --- | --- | --- | --- |
| TLEO | .101 | .305 | .063 |
| TLEC | .226 | .386 | .050 |
| TSEO | .232 | .525 | -.075 |
| TSEC | .303 | .521 | .004 |
| TLEOFP | .437 | .567 | .059 |
| TLECFP | .063 | .247 | .139 |
| TSEOFP | .404 | .128 | -.087 |
| TSECFP | .372 | .263 | -.042 |
| HT | -.021 | -.065 | .105 |
| TMB_Dual_Bal | .166 | .503 | -.016 |
| DSB_Bal | .452 | .694 | -.162 |
| TMA_COG | -.417 | -.331 | .222 |
| TMB_COG | -.242 | -.061 | -.021 |
| TMB_Dual_COG | -.494 | .267 | .160 |
| hsa-let-7b-3p | -.622 | .125 | .343 |
| hsa-miR-2682-5p | .347 | .009 | .846 |
| hsa-miR-3118 | .841 | -.322 | -.267 |
| hsa-miR-3170 | .731 | -.008 | -.221 |
| hsa-miR-3919 | .818 | -.102 | .517 |
| hsa-miR-433-3p | .683 | -.398 | .248 |
| hsa-miR-4632-3p | .900 | -.247 | -.239 |
| hsa-miR-4660 | .573 | .132 | .406 |
| hsa-miR-4760-5p | -.093 | -.279 | -.444 |
| hsa-miR-601 | .403 | -.300 | .368 |
| hsa-miR-608 | .131 | -.289 | .367 |
| hsa-miR-6870-3p | .815 | -.300 | -.346 |

**S2 Table. Factor weights from PCA of ASR miRNAs and functional data.**
