# Supplementary material for: Comparison of serum and saliva miRNAs for identification and characterization of mTBI in adult mixed martial arts fighters
Source: PLoS One. 2019 Jan 2;14(1):e0207785. doi: 10.1371/journal.pone.0207785 (PMC6314626; doi:10.1371/journal.pone.0207785)
Supplement: S3 Table — (DOCX) [file pone.0207785.s011.docx]

**S3 Table. Factor weights from PCA of ASR miRNAs and functional data.**

| **Measure** | **Factor 1** | **Factor 2** | **Factor 3** |
| --- | --- | --- | --- |
| TLEO | -.142 | .152 | -.036 |
| TLEC | -.167 | .128 | -.064 |
| TSEO | -.558 | .107 | .139 |
| TSEC | -.350 | .238 | .171 |
| TLEOFP | -.431 | .436 | -.038 |
| TLECFP | -.076 | .154 | -.283 |
| TSEOFP | -.174 | .292 | -.028 |
| TSECFP | -.388 | .425 | -.074 |
| HT | .198 | .372 | -.310 |
| TMB_Dual_Bal | -.639 | .015 | .113 |
| DSB_Bal | -.644 | .727 | .623 |
| TMA_COG | .315 | -.118 | -.351 |
| TMB_COG | -.203 | -.260 | -.114 |
| TMB_Dual_COG | -.350 | -.188 | -.389 |
| hsa-miR-1270 | .239 | -.316 | .316 |
| hsa-miR-139-5p | -.448 | -.531 | .321 |
| hsa-miR-30c-1-3p | -.328 | -.311 | .449 |
| hsa-miR-3664-3p | .446 | -.389 | .115 |
| hsa-miR-3678-3p | .552 | .270 | .198 |
| hsa-miR-421 | -.582 | -.363 | .336 |
| hsa-miR-4529-3p | .523 | -.160 | .570 |
| hsa-miR-4727-3p | .452 | .291 | -.045 |
| hsa-miR-501-3p | -.154 | -.017 | -.251 |
| hsa-miR-550a-3-5p | -.128 | .003 | .026 |
| hsa-miR-5588-5p | .571 | .107 | .422 |
| hsa-miR-6809-3p | .800 | .238 | .193 |
| hsa-miR-8089 | .353 | .486 | .220 |
