# Supplementary material for: Comparison of serum and saliva miRNAs for identification and characterization of mTBI in adult mixed martial arts fighters
Source: PLoS One. 2019 Jan 2;14(1):e0207785. doi: 10.1371/journal.pone.0207785 (PMC6314626; doi:10.1371/journal.pone.0207785)
Supplement: S4 Table — Upper indicates exact miRNA matches in previous studies. Lower indicates highly-related miRNA matches in previous studies. HTH, changes related to hits to the head in current study; T, time-course changes in current study. We note that miR-155-5p was decreased in severe TBI as determined by microarray analysis in one of the studies, but failed to show differential expression in qRT-PCR validation assay. Similarly, miR-455-3p was decreased in mild TBI as determined by microarray analysis, but failed to show differential expression in qRT-PCR validation assay. (DOCX) [file pone.0207785.s012.docx]

**S4 Table.**  **miRNAs with significant effect of HTH (Table 5) or defined temporal effects (S1 Table) that have been previously reported in TBI studies.**

**Exact miRNA matches in previous studies:**

| **miRNA** | **Change** | **TBI Severity** | **Fluid/Tissue** | **Species** | **Ref** |
| --- | --- | --- | --- | --- | --- |
| hsa-miR-122-5p^HTH^ | ↑ | mild | serum | rat | [30] |
| hsa-miR-128-3p^HTH^ | ↑ | mild | saliva | human | [13] |
|  | ↑ | mild, mild-moderate | plasma | mouse | [31] |
| hsa-miR-139-5p^T^ | ↓ | mild-moderate | dentate gyrus | rat | [32] |
| hsa-miR-421^T^ | ↓ | mild | Serum  saliva | Mouse  human | [33]  [13] |
| hsa-miR-433-3p^T^ | ↓ | moderate | hippocampus | rat | [34] |
| hsa-miR-601^T^ | ↑ | severe | serum | human | [35] |
| hsa-1307-3p^HTH^ | ↑ | mild | saliva | human | [13] |

**Related miRNA matches in previous studies:**

| **miRNA** | **Related miRNA** | **Change** | **TBI Severity** | **Fluid/Tissue** | **Species** | **Ref** |
| --- | --- | --- | --- | --- | --- | --- |
| hsa-let-7b-3p^T^ | let-7b | ↓ | mild-moderate | hippocampus | rat | [36] |
|  | let-7b-5p | ↓ | mild | saliva | human | [13] |
| hsa-miR-20a-5p^HTH^ | miR-20a | ↑ | mild, moderate, severe | serum | human | [37] |
| hsa-miR-30b-5p^HTH^ | miR-30b | ↑ | moderate | hippocampus | rat | [34] |
|  | miR-30b | ↑ | severe | CSF | human | [37] |
| hsa-miR-30c-1-3p^T^ | miR-30c-1 | ↓ | mild | saliva | human | [13] |
| hsa-miR-92a-3p^HTH^ | miR-92a | ↑ | mild | plasma | human | [38] |
|  | miR-92a | ↓ | severe | plasma | human | [38] |
| hsa-miR-155-5p^HTH^ | miR-155 | ↑ | moderate | hippocampus | rat  mouse | [34]  [39] |
| hsa-miR-376a-5p^HTH^ | miR-376a | ↑ | mild | serum | mouse | [33] |
|  | miR-376a | ↓ | mild-moderate | dentate gyrus | rat | [32] |
|  | miR-376a | ↓ | moderate | hippocampus | rat | [34] |
|  | miR-376a* | ↑ | mild | parietal lobe | mouse | [40] |
| hsa-miR-455-5p^HTH^ | miR-455-3p | ↓ | mild | PBMCs | human | [41] |
|  | miR-455 | ↑ | mild | serum | mouse | [33] |
| hsa-miR-501-3p^T^ | miR-501 | ↓ | mild | saliva | human | [13] |

Upper indicates exact miRNA matches in previous studies. Lower indicates highly-related miRNA matches in previous studies. HTH, changes related to hits to the head in current study; T, time-course changes in current study. We note that miR-155-5p was decreased in severe TBI as determined by microarray analysis in one of the studies, but failed to show differential expression in qRT-PCR validation assay. Similarly, miR-455-3p was decreased in mild TBI as determined by microarray analysis, but failed to show differential expression in qRT-PCR validation assay.
